# Supplementary material for: Protective effects of macrophage-specific integrin α5 in myocardial infarction are associated with accentuated angiogenesis
Source: Nat Commun. 2023 Nov 20;14:7555. doi: 10.1038/s41467-023-43369-x (PMC10662477; doi:10.1038/s41467-023-43369-x)
Supplement: Supplementary file 1 — Supplementary Information [file 41467_2023_43369_MOESM1_ESM.pdf]

Protective effects of macrophage-specific integrin α5 in myocardial infarction are associated with accentuated angiogenesis

Ruoshui Li[1,2], Bijun Chen[1,2], Akihiko Kubota[1,2], Anis Hanna[1,2], Claudio Humeres[1,2], Silvia C Hernandez[1,2], Yang Liu[3], Richard Ma[3], Izabela Tuleta[1,2], Shuaibo Huang[1,2], Harikrishnan Venugopal[1,2], Fenglan Zhu[1,2], Kai Su[1,2], Jun Li [1,2], Jinghang Zhang[2], Deyou Zheng[3,4,5], and Nikolaos G Frangogiannis[1.2].

[1]The Wilf Family Cardiovascular Research Institute, Department of Medicine (Cardiology), [2]Department of Microbiology and Immunology, [3]Department of Genetics, [4]Department of Neurology, and [5]Department of Neuroscience, Albert Einstein College of Medicine, Bronx NY, USA.
.

ONLINE SUPPLEMENT

SUPPLEMENTAL FIGURES:

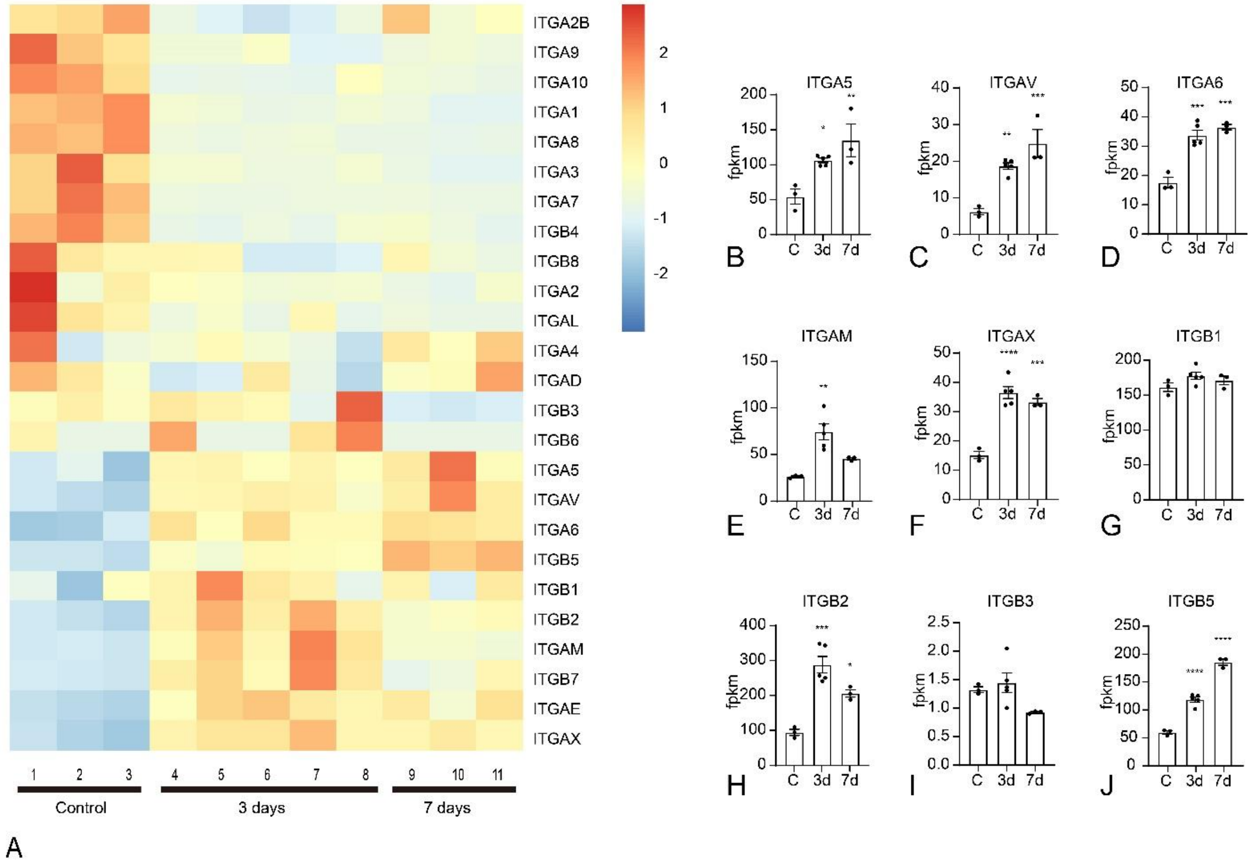

Supplemental Figure 1: Integrin expression profile of infarct macrophages. A: A heatmap of our RNA-seq data illustrates the integrin expression profile in CD11b+/Ly6G- macrophages harvested from control and infarcted hearts (3-7 days after coronary occlusion). Each column represents one mouse. B-J: Quantitative analysis of gene expression of specific integrin chains. Infarct macrophages had a marked upregulation of *Itga5* (B), *Itgav* (C) and *Itga6* (D) after 3 and 7 days of coronary occlusion. *Itgam* expression (E) peaked at the 3-day timepoint, whereas *Itgax* levels (F) increased after 3 and 7 days of coronary occlusion. *Itgb1* expression (G) was high in control macrophages and did not significantly increase after 3-7 days of coronary ischemia. *Itgb2* was highly expressed in control cardiac macrophages, and increased markedly after 3-7 days (H). In contrast, *Itgb3* expression levels (I) were low in control and infarct macrophages. *Itgb5* (J) exhibited progressive increase in infarct macrophages after 3-7 days of coronary occlusion (Data are shown as mean values +/- SEM. ****$p<0.0001$, ***$p<0.001$, **$p<0.01$, *$p<0.05$, n=3 biologically independent experiments in C and 7d group, n = 5 biologically independent experiments in 3d group). For statistical analysis one-way ANOVA followed by Sidak post-hoc test was used. Source data are provided as a Source Data file.

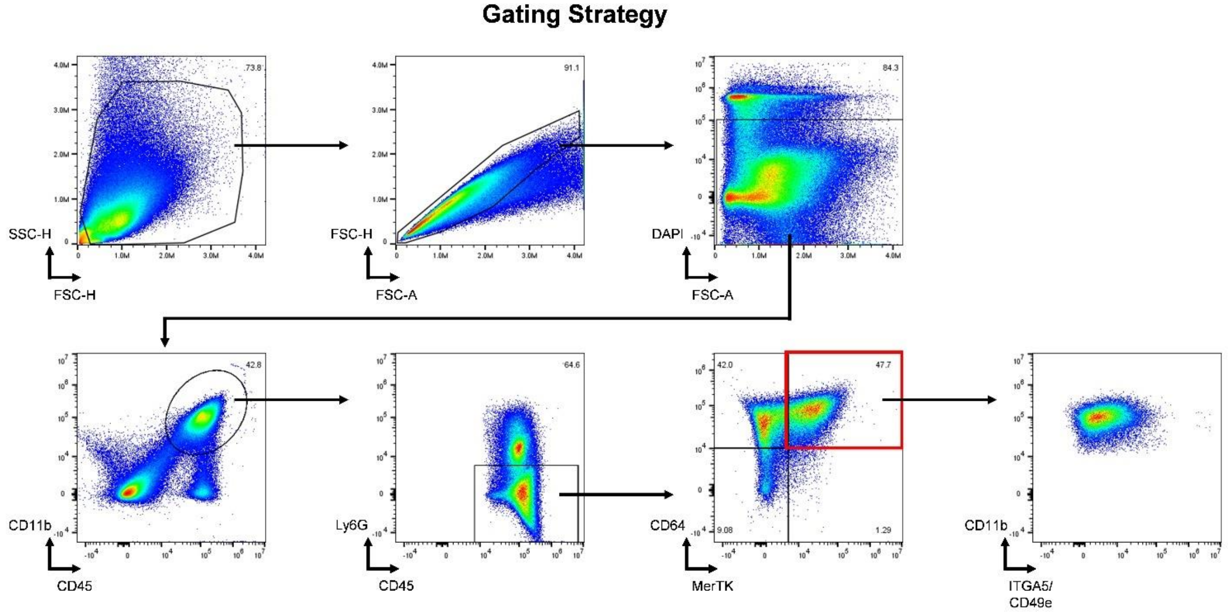

Supplemental Figure 2: Gating strategy for flow cytometry experiments assessing the time course of ITGA5 expression in infarct macrophages. Representative images show flow cytometric analysis of single cell cardiac suspensions from an infarcted mouse heart (7 days coronary occlusion). After doublets were excluded by FSC-H vs. FSC-A, DAPI-negative live cells were identified. Myeloid cells were identified as DAPI- CD45+ CD11b+ cells. After exclusion of DAPI- CD45+ CD11b+ Ly6G+ cells (neutrophils), macrophages were identified as DAPI- CD45+ CD11b+ Ly6G- CD64+ MerTK+ cells. Expression of ITGA5/CD49e was assessed in macrophages. FSC: forward scatter; SSC: side scatter. Representative images from the experiments illustrated in Figure 1A-C (Sham: n=5 biologically independent experiments, 7-day: n=8 biologically independent experiments, 28-day: n=11 biologically independent experiments)

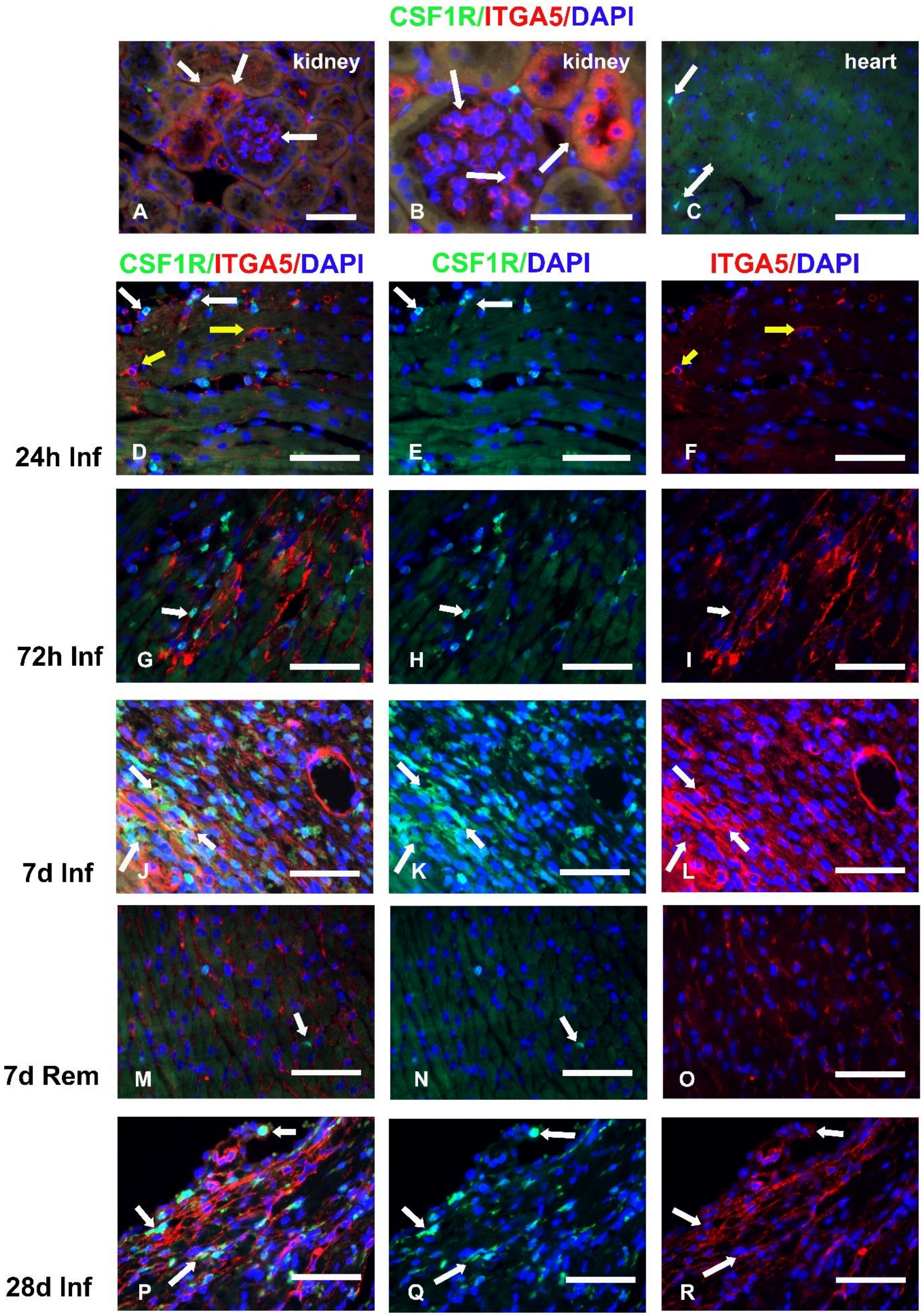
CSF1R/ITGA5/DAPI
kidney
kidney
heart
A
B
C
CSF1R/ITGA5/DAPI
CSF1R/DAPI
ITGA5/DAPI
24h Inf
D
E
F
72h Inf
G
H
I
7d Inf
J
K
L
7d Rem
M
N
O
28d Inf
P
Q
R

Supplemental Figure 3: Time course of ITGA5 immunoreactivity in infarct macrophages. $CSF1R^{EGFP}$ macrophage reporter mice underwent non-reperfused infarction protocols. Representative images of infarcted areas are shown after 24h (D-F), 72h (G-I), 7 days (J-L) and 28 days (P-R) of coronary occlusion. Sections from the mouse kidney serve as positive controls, showing staining in glomerular and tubular cells (A-B, arrows). In contrast, CSF1R+ macrophages in normal hearts have negligible ITGA5 immunoreactivity (C, arrows). 24h after infarction, most CSF1R+ macrophages had low level ITGA5 expression (D-F, white arrows). At this timepoint, ITGA5 was predominantly localized in CSF1R-negative cells with morphological characteristics of vascular cells or interstitial fibroblasts (D-F, yellow arrows). Dual immunofluorescence for GFP (CSF1R labeling) and for ITGA5 showed expansion of the ITGA5+ macrophage population 7 days after myocardial infarction (J-L, arrows). Several ITGA5+/CSF1R+ cells were noted 28 days after infarction (P-R, arrows). Macrophages in remote non-infarcted myocardium had negligible ITGA5 immunofluorescence (M-O, arrow). Representative images of the experiments shown in Figure 1D-K (n=6 biologically independent experiments in C, 24-hour, 7-day and 28-day groups, n=5 biologically independent experiments in 3-day group). Quantitative analysis is shown in Figure 1K. Scalebar= 60μm. Inf: infarct area; Rem: remote area.

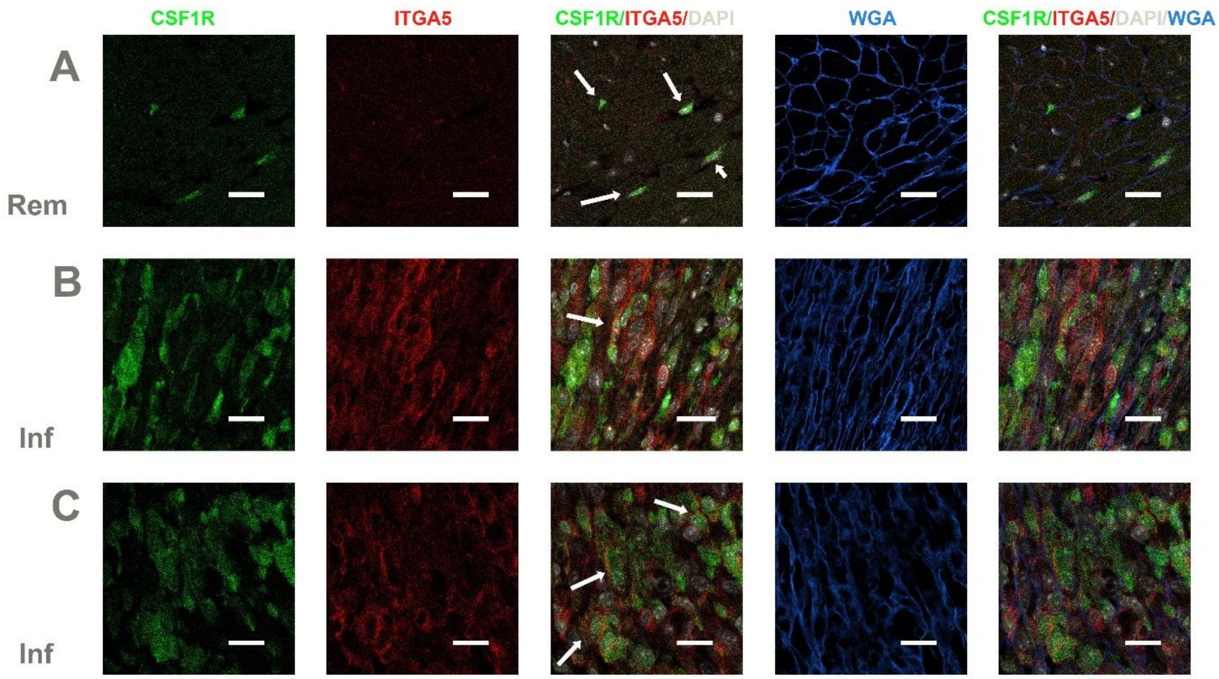

Supplemental Figure 4: ITGA5 localization on the cell membrane and in the cytoplasm of infarct macrophages. CSF1R^{EGFP} macrophage reporter mice underwent non-reperfused myocardial infarction protocols (7 days coronary occlusion). Sections from infarcted (Inf, B-C) and remote non-infarcted myocardium (Rem, A) were stained for GFP (to identify CSF1R+ macrophages), ITGA5, WGA (to label membrane and ECM glycoproteins) and DAPI. Representative confocal microscopy images show negligible ITGA5 immunoreactivity in macrophages in non-infarcted areas (A, arrows). ITGA5 immunoreactivity is noted in infarct macrophages and is localized predominantly on the cell surface (C, arrows). However, several macrophages also exhibit cytoplasmic ITGA5 staining (B, arrow), which likely reflects de novo synthesis of ITGA5 that is subsequently shuttled to the cell membrane. Representative images were selected from 50 different scanned fields from 6 biologically independent experiments. Scalebar=10μm. Inf: infarct area; Rem: remote area.

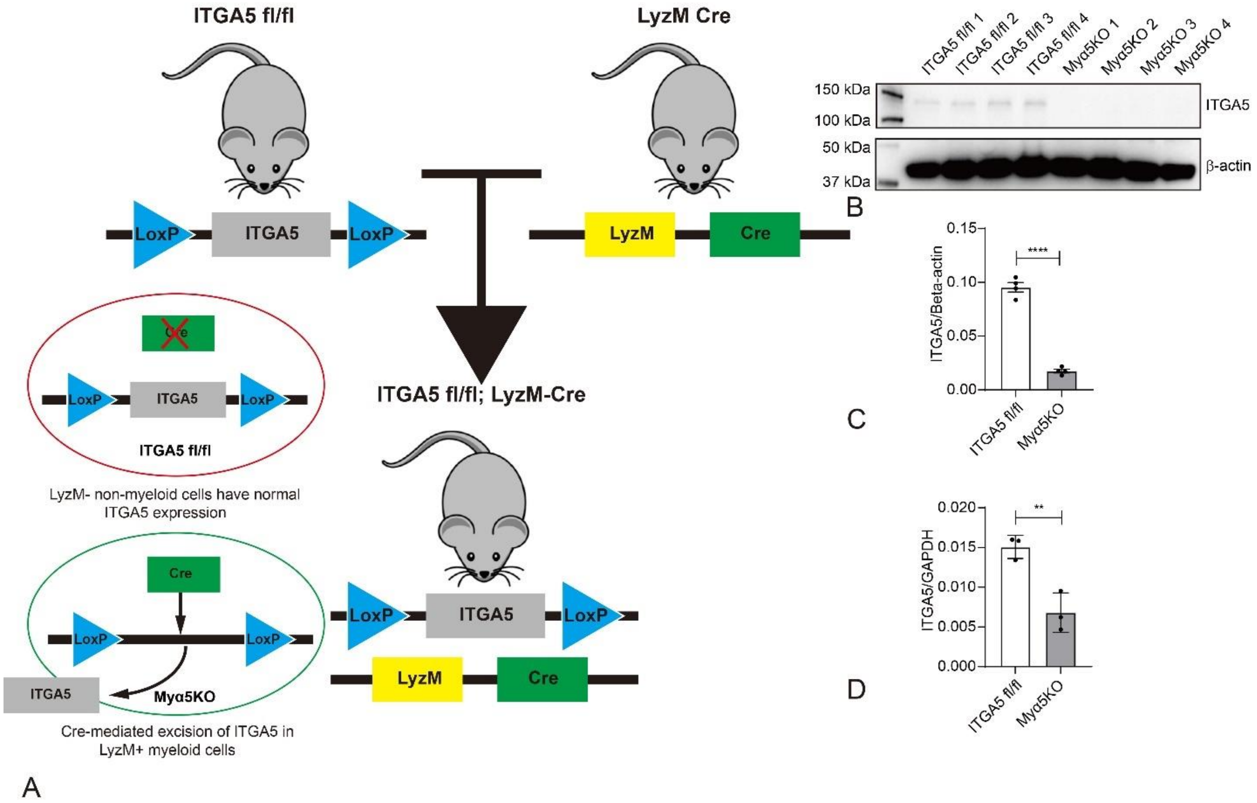

Supplemental Figure 5: Generation and validation of mice with myeloid cell-specific ITGA5 deletion. A: Schematic representation of the breeding strategy utilized to generate myeloid cell specific ITGA5 knockout mice (Myα5KO) by Cre-mediated excision of ITGA5 in LyzM-expressing myeloid cells. B-C: Representative images of western blotting experiments (B), followed by quantitative analysis (C) show that bone-morrow macrophages isolated from Myα5KO mice exhibit marked reduction in ITGA5 expression levels (****p<0.0001, n=4 biologically independent experiments/group). D: qPCR shows that *Itga5* expression is markedly reduced in CD11b+/Ly6G- macrophages isolated from Myα5KO infarcted hearts (after 7 days of coronary occlusion) in comparison to macrophages harvested from ITGA5 fl/fl infarcts (**p<0.01, n=3 biologically independent experiments/group). Data are shown as mean values +/- SEM. Statistical analysis was performed using unpaired two-tailed Student’s t test. Source data are provided as a Source Data file.

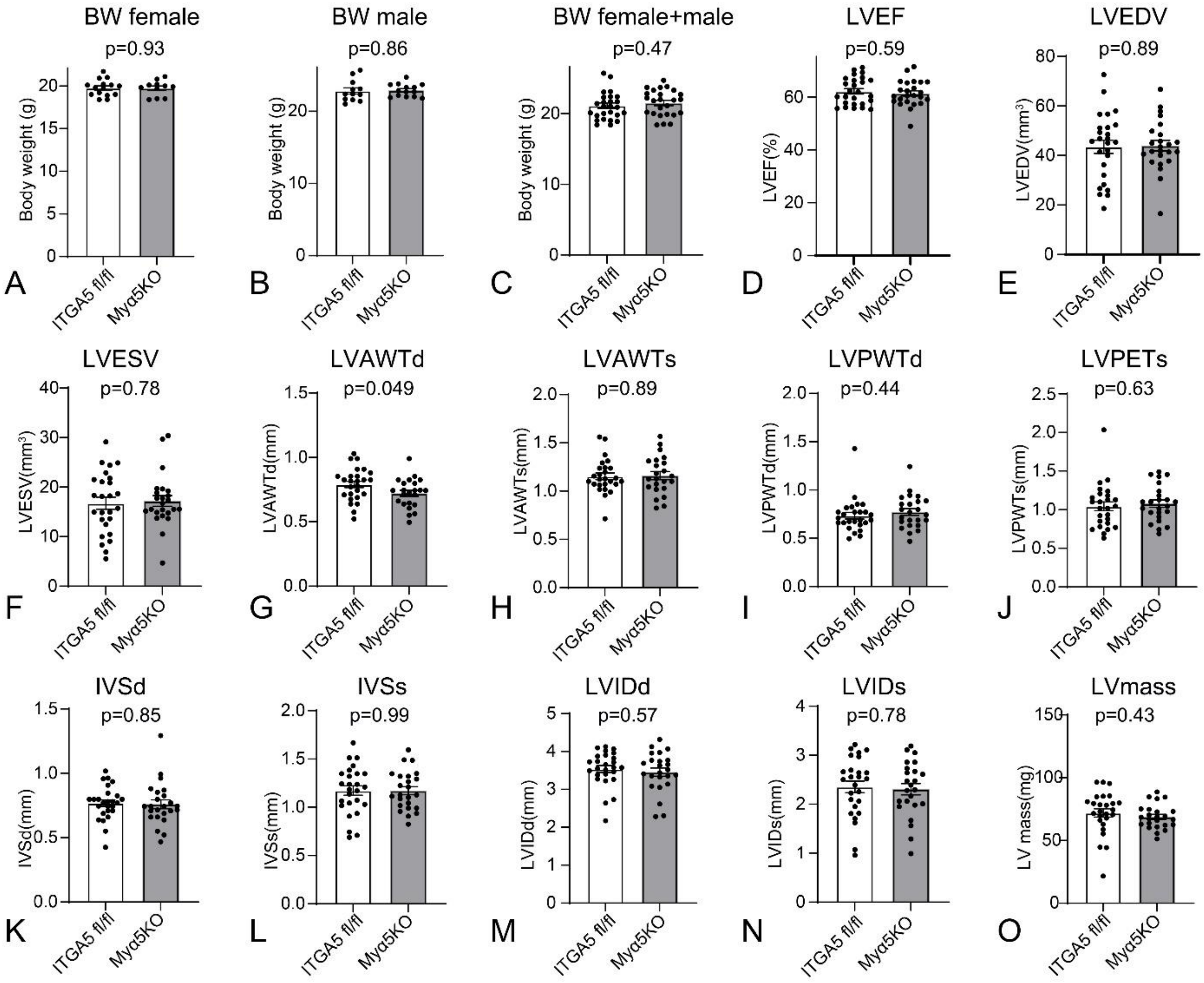

Supplemental Figure 6: Myeloid cell-specific ITGA5 loss does not affect body weight, cardiac dimensions and function in young adult mice. A-C: Myα5KO and ITGA5 fl/fl controls had comparable body weight (BW). D-O: Echocardiographic analysis showed that myeloid cell-specific ITGA5 loss did not affect baseline cardiac geometry and function. ITGA5 fl/fl mice and Myα5KO animals had no significant baseline differences in left ventricular ejection fraction (LVEF; D), left ventricular end-diastolic volume (LVEDV; E), left ventricular end-systolic volume (LVESV; F), left ventricular anterior wall systolic thickness (LVAWTs; H), left ventricular posterior wall diastolic thickness (LVPWTd; I), left ventricular posterior wall systolic thickness (LVPWTs; J), interventricular septal diastolic thickness (IVSd; K), interventricular septal systolic thickness (IVSs; L), left ventricular internal diastolic diameter (LVIDd; M), left ventricular internal systolic diameter (LVIDs; N), and left ventricular mass (LV mass, O). A modest (<10%) reduction in left ventricular anterior wall end-diastolic thickness (LVAWTd; G) was noted in Myα5KO mice that barely reached statistical significance (p=0.049) but was not accompanied by effects on LV mass or septal/posterior wall thickness. (Data are shown as mean values +/- SEM. For panel A: ITGA5 fl/fl: n=15, Myα5KO: n=11 biologically independent experiments; For B: ITGA5 fl/fl: n=12, Myα5KO: n=14 biologically independent experiments; For C: ITGA5 fl/fl:

n=27, Myα5KO: n=25 biologically independent experiments; For G and H: ITGA5 fl/fl: n=26, Myα5KO: n=23 biologically independent experiments. For the other panels: ITGA5 fl/fl: n=26, Myα5KO: n=24 biologically independent experiments). Unpaired two-tailed Student's t test was used to compare the differences between the groups. Source data are provided as a Source Data file.

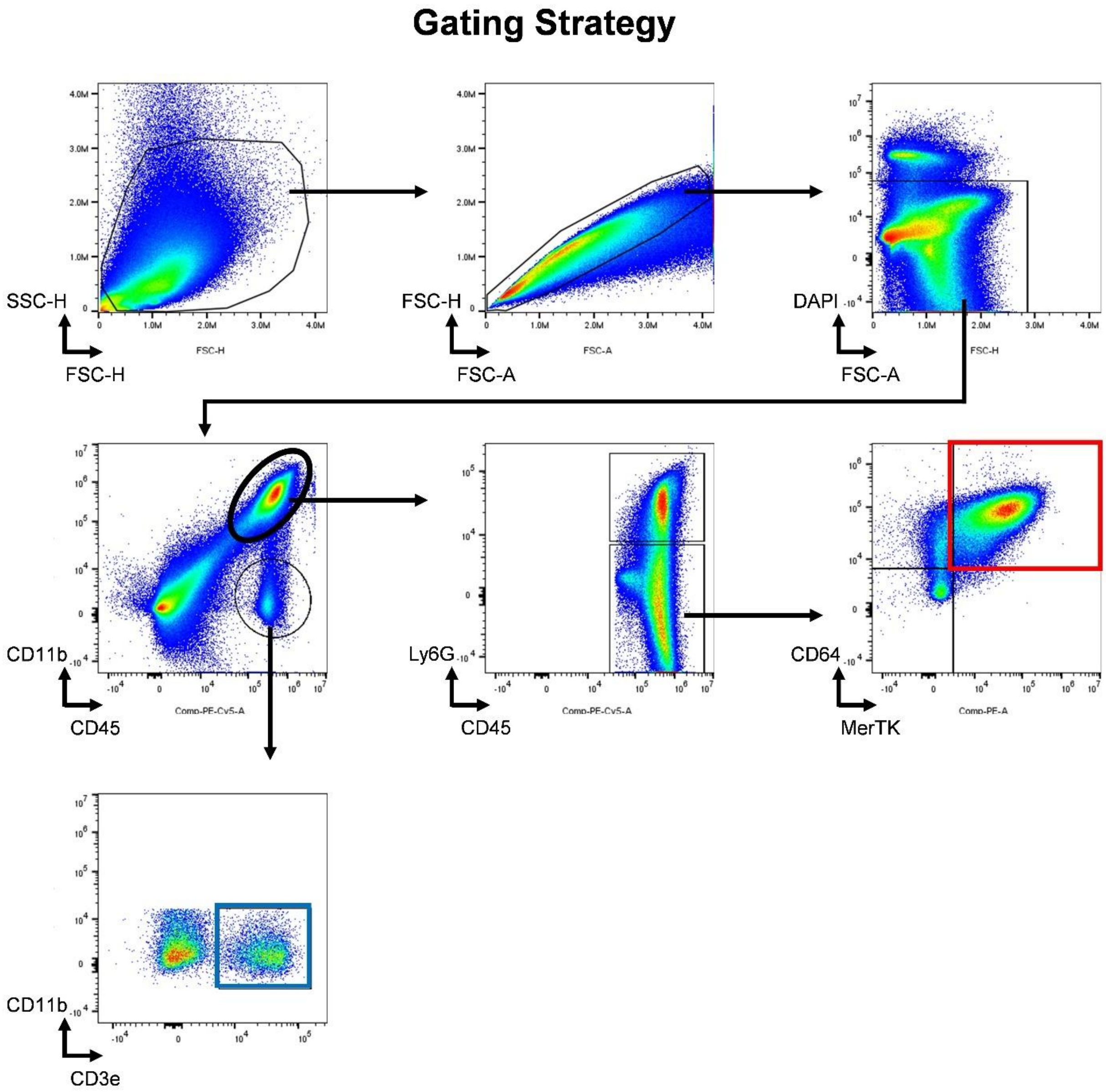

Supplemental Figure 7: Gating strategy for flow cytometry experiments comparing infiltration of Myα5KO and ITGA5 fl/fl infarcts with myeloid cells, macrophages and T cells. Representative images show flow cytometric analysis of single cell cardiac tissue suspensions from an infarcted Myα5KO mouse (after 7 days of coronary occlusion). After doublets were excluded by FSC-H vs. FSC-A, DAPI-negative live cells were identified. Myeloid cells were identified as DAPI- CD45+ CD11b+ cells. After exclusion of DAPI- CD45+ CD11b+ Ly6G+ cells (neutrophils) among myeloid cells, macrophages were identified as DAPI- CD45+ CD11b+ Ly6G- CD64+ MerTK+ cells. T cells were identified as DAPI- CD45+ CD11b- CD3e+ cells. FSC: forward scatter; SSC: side scatter. Representative images from the experiments illustrated in Figure 4. (5 biologically independent experiments/group).

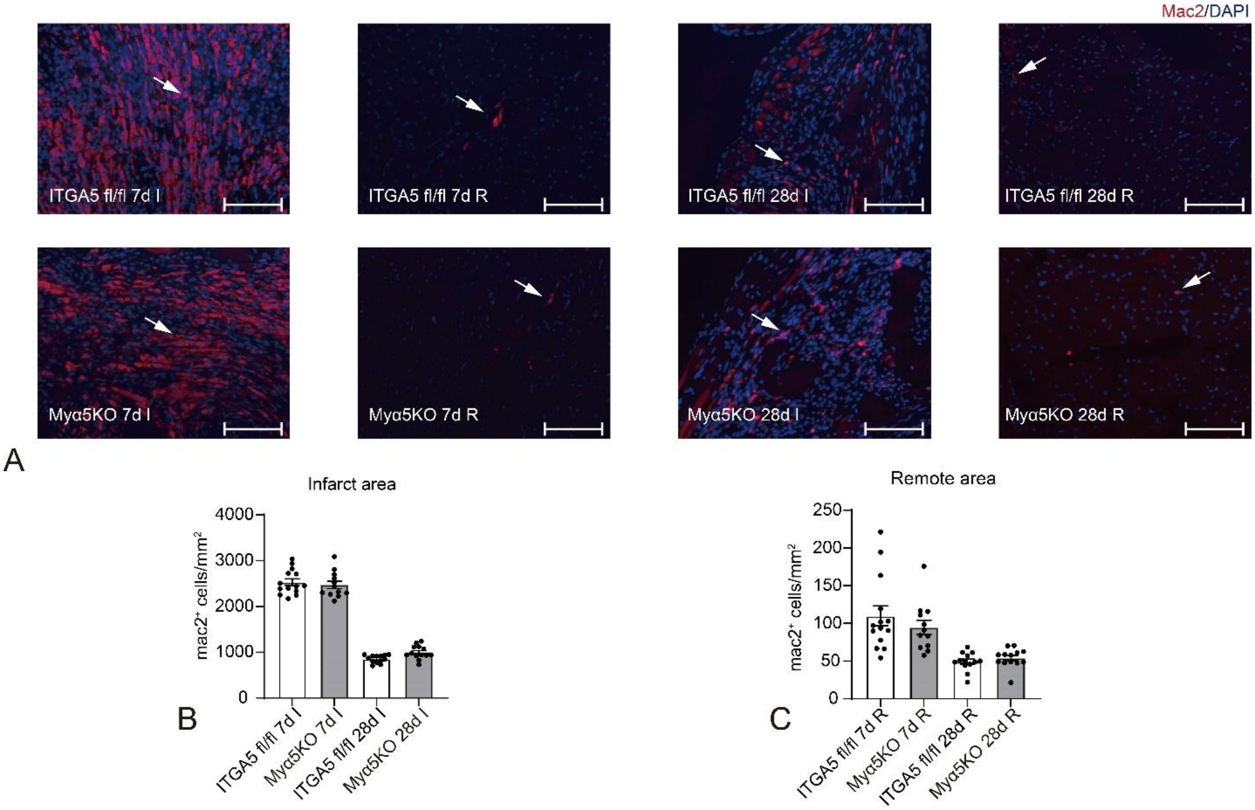

Supplemental Figure 8: Myeloid cell-specific ITGA5 loss does not affect infiltration of the infarcted myocardium with macrophages. Infarct macrophages were identified in ITGA5 fl/fl and in Myα5KO infarcts (A) using immunofluorescence for Mac2 (red fluorescence - arrows). In comparison to the marked expansion of macrophages in the infarcted heart, the number of macrophages in the remote remodeling myocardium remained low. Quantitative analysis showed that Myα5KO mice and corresponding ITGA5 fl/fl animals had comparable macrophage density in the infarct and in the remote remodeling myocardium(B-C). Scalebar=100μm. (Data are shown as mean values +/- SEM. ITGA5 fl/fl: n=14 biologically independent experiments in 7d group, n=13 biologically independent experiments in 28d group; Myα5KO: n=12 biologically independent experiments in 7d group, n=13 biologically independent experiments in 28d group). One-way ANOVA and post hoc Sidak test were used to compare differences between the groups. Source data are provided as a Source Data file. I: infarct area; R: remote area.

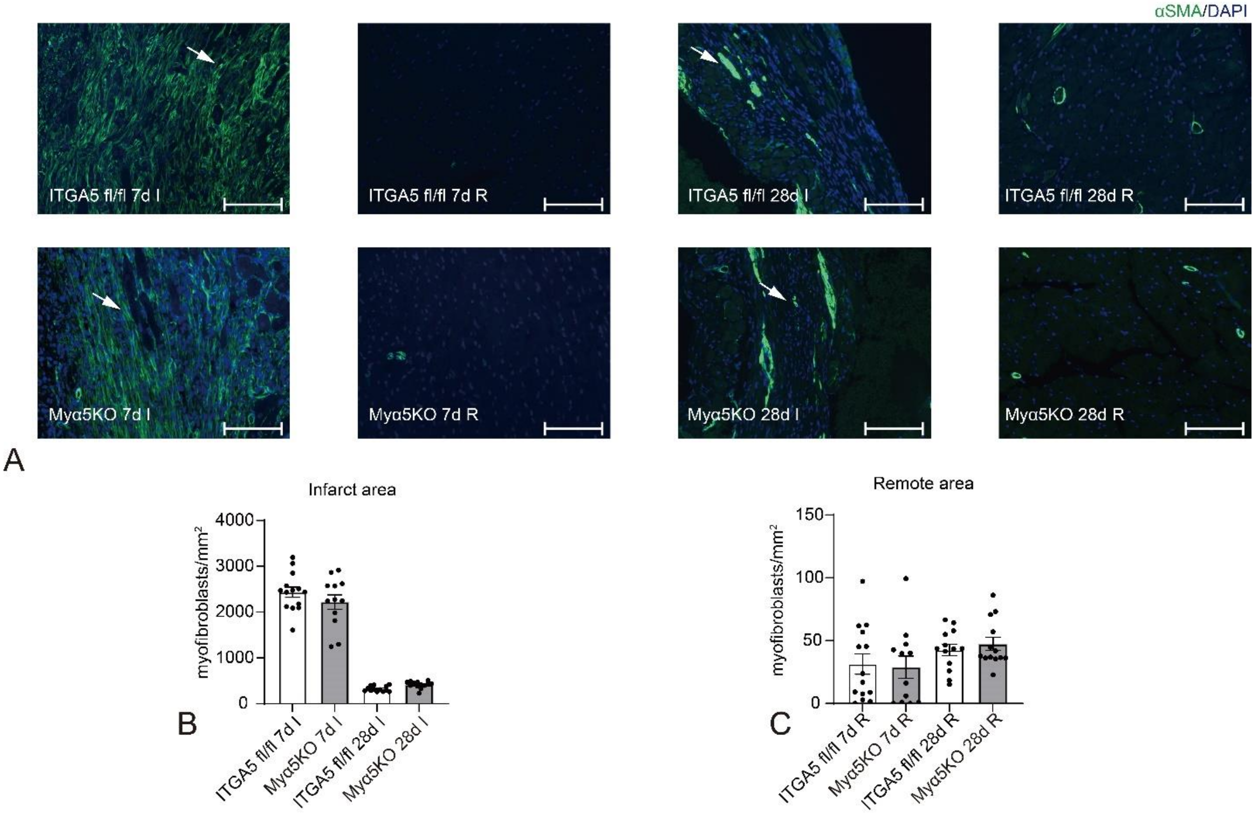

Supplemental Figure 9: Myeloid cell specific ITGA5 loss does not affect the time course of myofibroblast infiltration in the healing infarct. Infarct myofibroblasts were identified in ITGA5 fl/fl and in Myα5KO infarcts (A) as α-smooth muscle actin (α-SMA)+ cells located outside the vascular media (green fluorescence - arrows) In contrast to the marked expansion of myofibroblasts in the infarct, the number of myofibroblast in the remote remodeling myocardium was low. Quantitative analysis showed that Myα5KO mice and corresponding ITGA5 fl/fl animals had comparable myofibroblast density in the infarct and in the remote remodeling myocardium (B-C). Scalebar=50μm. Data are shown as mean values +/- SEM. ITGA5 fl/fl: n=14 biologically independent experiments in 7d group, n=13 biologically independent experiments in the 28d group; Myα5KO: n=12 biologically independent experiments in the 7d group, n=13 biologically independent experiments in the 28d group. One-way ANOVA and post hoc Sidak tests were used to compare differences between the groups. Source data are provided as a Source Data file. I: infarct area; R: remote area.

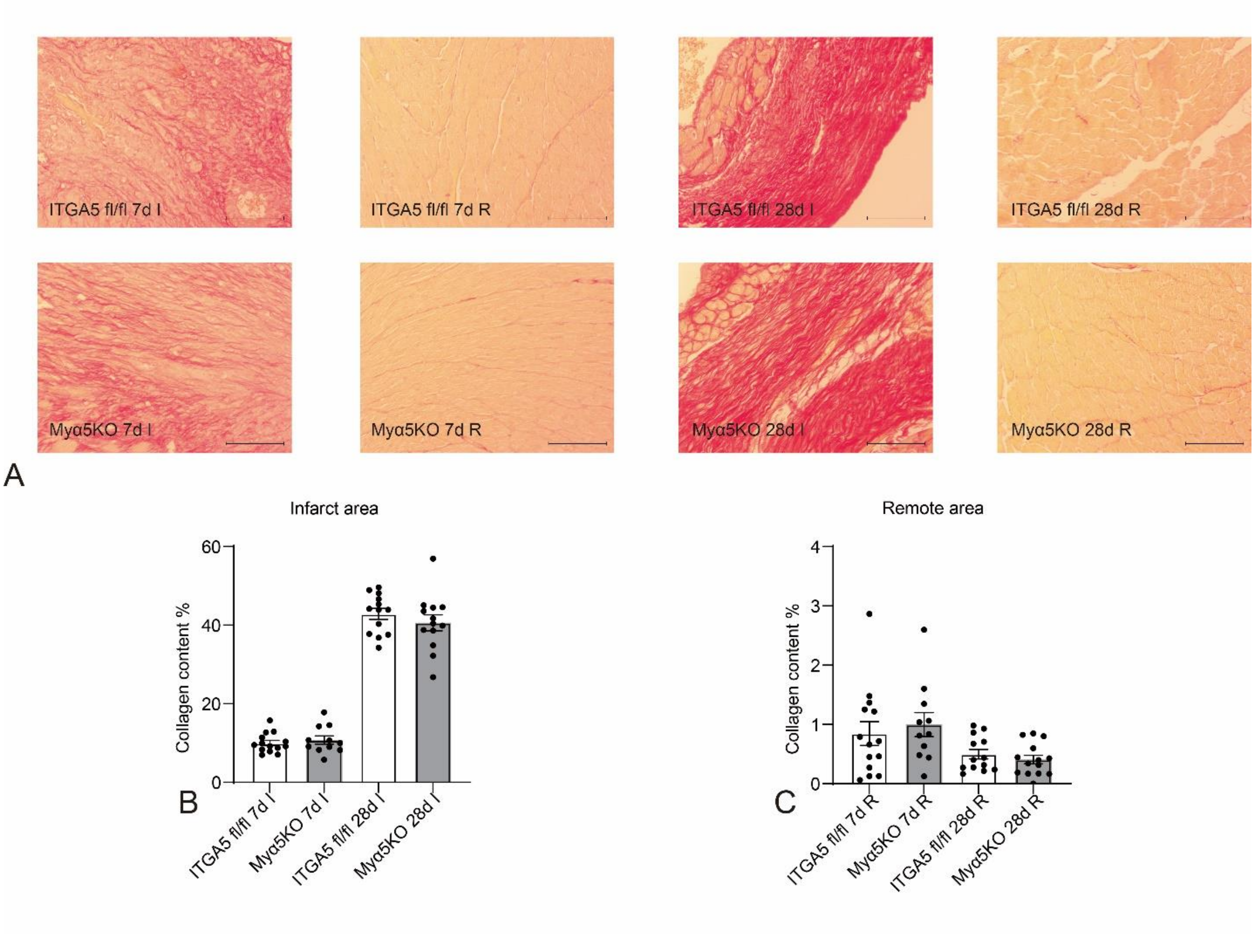

Supplemental Figure 10: Myeloid cell-specific ITGA5 loss does not have significant effects on collagen content in the infarct zone and in the remote remodeling myocardium. Picrosirius red staining was used to label collagen fibers in the infarcted and in the remote remodeling myocardium (A) of ITGA5 fl/fl and in Myα5KO mice after 7-28 days of coronary occlusion. Quantitative analysis showed no significant effects of myeloid cell-specific ITGA5 loss on collagen content in the infarct zone and in the remote remodeling myocardium (B-C). Scalebar=100µm. (Data are shown as mean values +/- SEM. ITGA5 fl/fl: n=14 biologically independent experiments in the 7d group, n=13 biologically independent experiments in the 28d group; Myα5KO: n=11 biologically independent experiments in the 7d group, n=13 biologically independent experiments in the 28d infarct group, n=14 biologically independent experiments in the 28d remote group). One-way ANOVA and post hoc Sidak tests were used to compare differences between groups. Source data are provided as a Source Data file. I: infarct area; R: remote area.

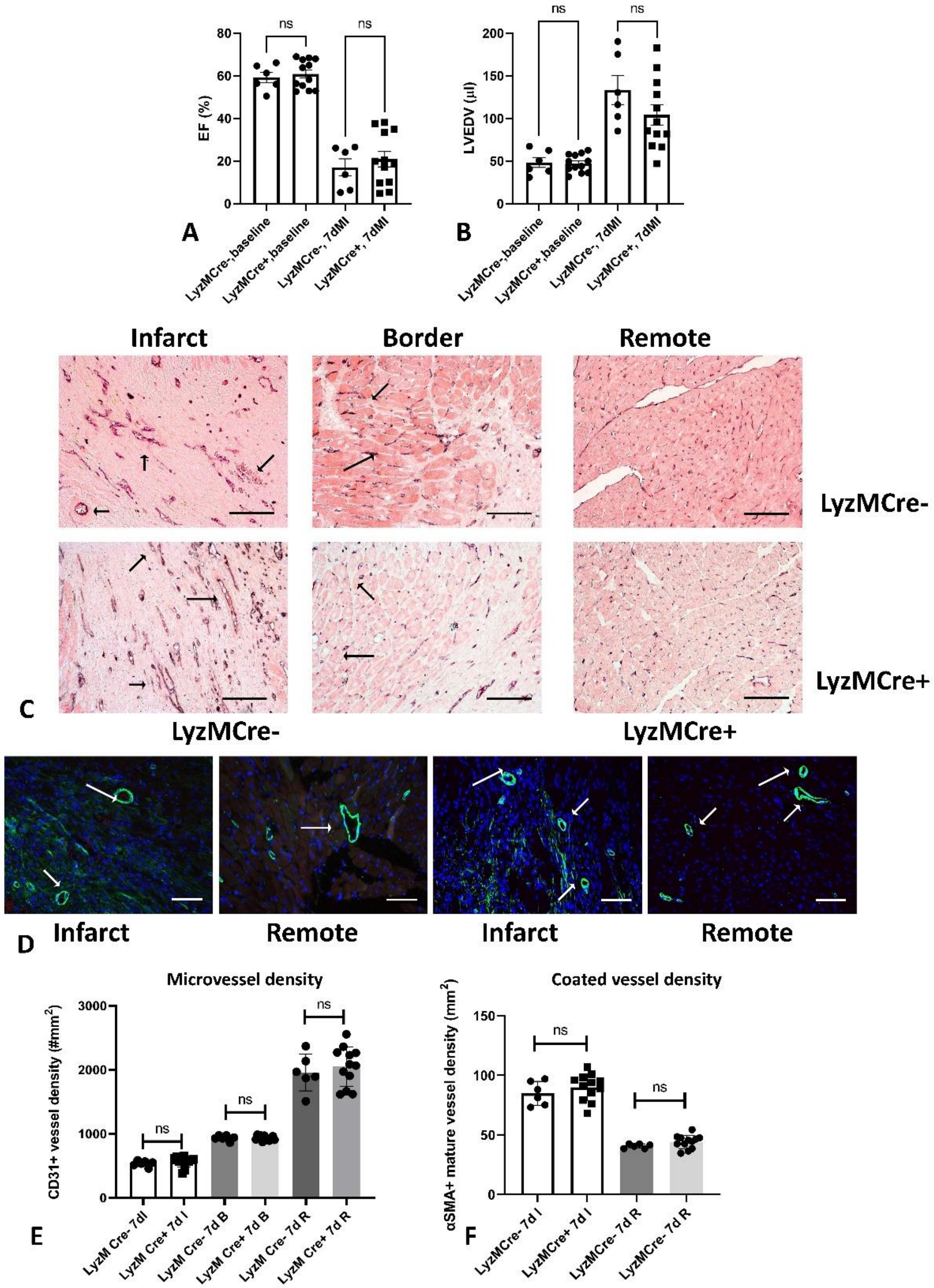

Supplemental Figure 11: Cre expression in Lysozyme M-Cre animals has no impact on post-infarction systolic dysfunction, adverse remodeling and infarct angiogenesis. In order to exclude that the enhanced dysfunction and perturbed angiogenesis, noted in LyzMCre;ITGA5fl/fl mice 7 days after infarction, are due to Cre expression (rather than ITGA5 loss),we compared function and infarct angiogenesis between LyzM-Cre animals and corresponding Cre-negative

controls. Ejection fraction (EF, A) and LVEDV (B) were comparable between groups (LyzMCre-, n=6 biologically independent experiments; LyzMCre+, n=12 biologically independent experiments). Microvessels in the infarct zone, border zone and remote remodeling myocardium were labeled using CD31 immunohistochemistry (arrows, C), whereas the density of mature coated vessels (D) was assessed using α-SMA immunofluorescence (arrows). Quantitative analysis showed that Cre expression did not affect microvascular density (E) and the number of mature coated vessels (F). LyzMCre-, n=6 biologically independent experiments; LyzMCre+, n=12 biologically independent experiments. Scalebar=80μm. Data are shown as mean values +/- SEM. Statistical comparisons between groups were performed using one-way ANOVA followed by the Sidak post-hoc test. Source data are provided as a Source Data file. NS: no significance.

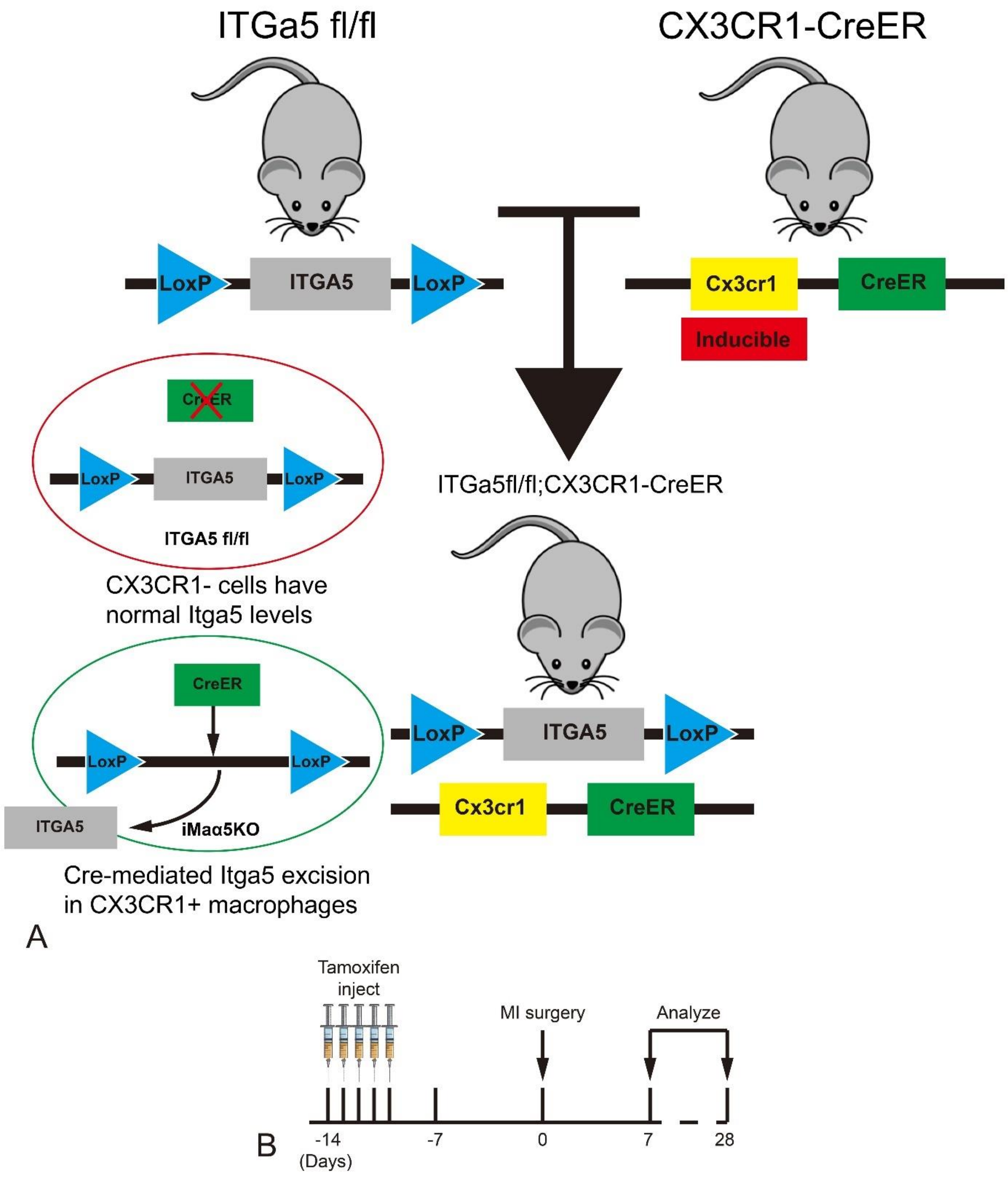

Supplemental Figure 12: Generation of inducible macrophage-specific ITGA5 KO mice (iMaα5KO**) using the CX3CR1-CreER driver.** A: Schematic representation of the breeding strategy utilized to generate iMaα5KO by Cre-mediated excision of ITGA5 in CX3CR1+ macrophages. B: Tamoxifen (100 mg/kg/day) was administered intraperitoneally every 24 h for 5

consecutive days (2 weeks before the coronary occlusion surgery) to generate mice with inducible ITGA5 loss in macrophages. MI: myocardial infarction.

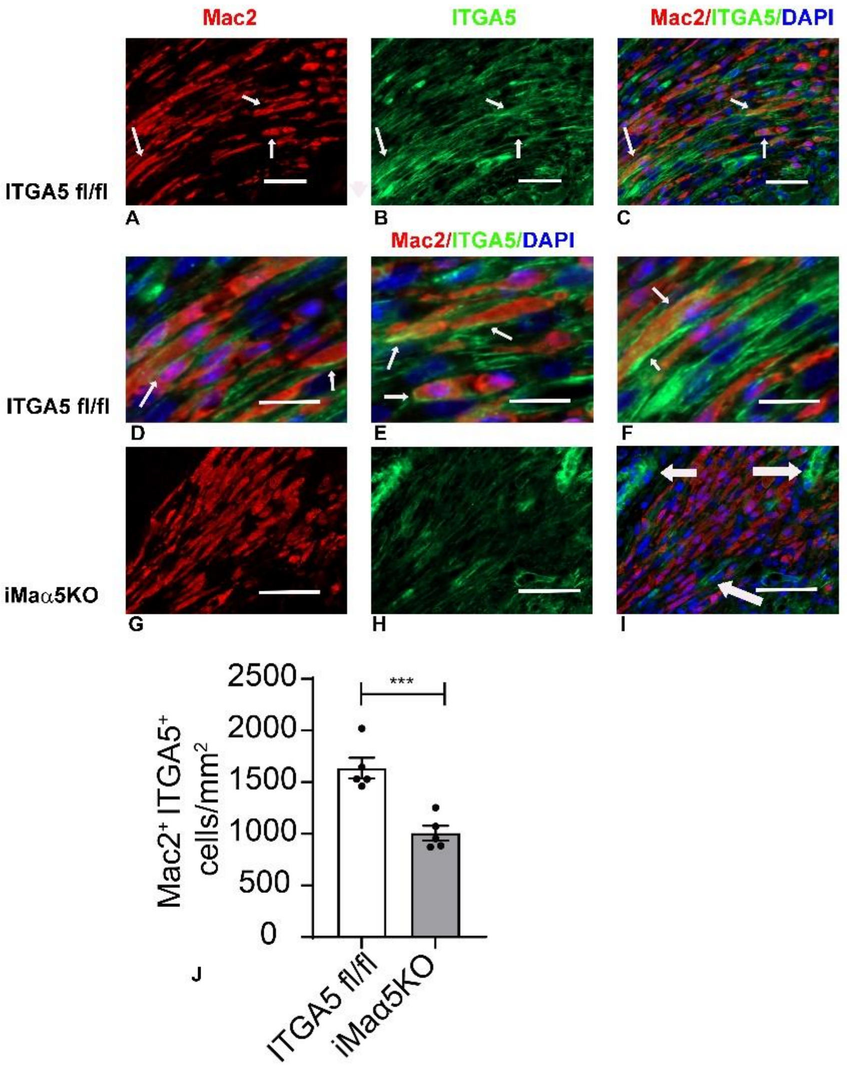

Supplemental Figure 13: Demonstration of macrophage-specific ITGA5 loss in iMaα5KO mice. Representative images show dual immunofluorescence for the macrophage marker Mac2 and ITGA5 in 7-day infarcts. A-F: In ITGA5 fl/fl mice, ITGA5 immunoreactivity is noted in Mac2+ macrophages and is predominantly localized on the cell surface (arrows). G-I: In macrophage-specific ITGA5 KO mice (iMaα5KO), Mac2+ macrophages have low levels of ITGA5 staining and ITGA5 immunofluorescence is localized in Mac2-negative cells with morphological characteristics of vascular and interstitial cells (I, arrows). J: Quantitative analysis showed a significant reduction in the number of Mac2+/ITGA5+ double positive cells in iMaα5KO infarcts, in comparison to their number in ITGA5 fl/fl infarcts. (***$p<0.001$, n=5 biologically independent experiments/group). Data are shown as mean values +/- SEM. Statistical analysis was performed using unpaired two-tailed Student's t test. Source data are provided as a Source Data file. Scalebar=30 μm.

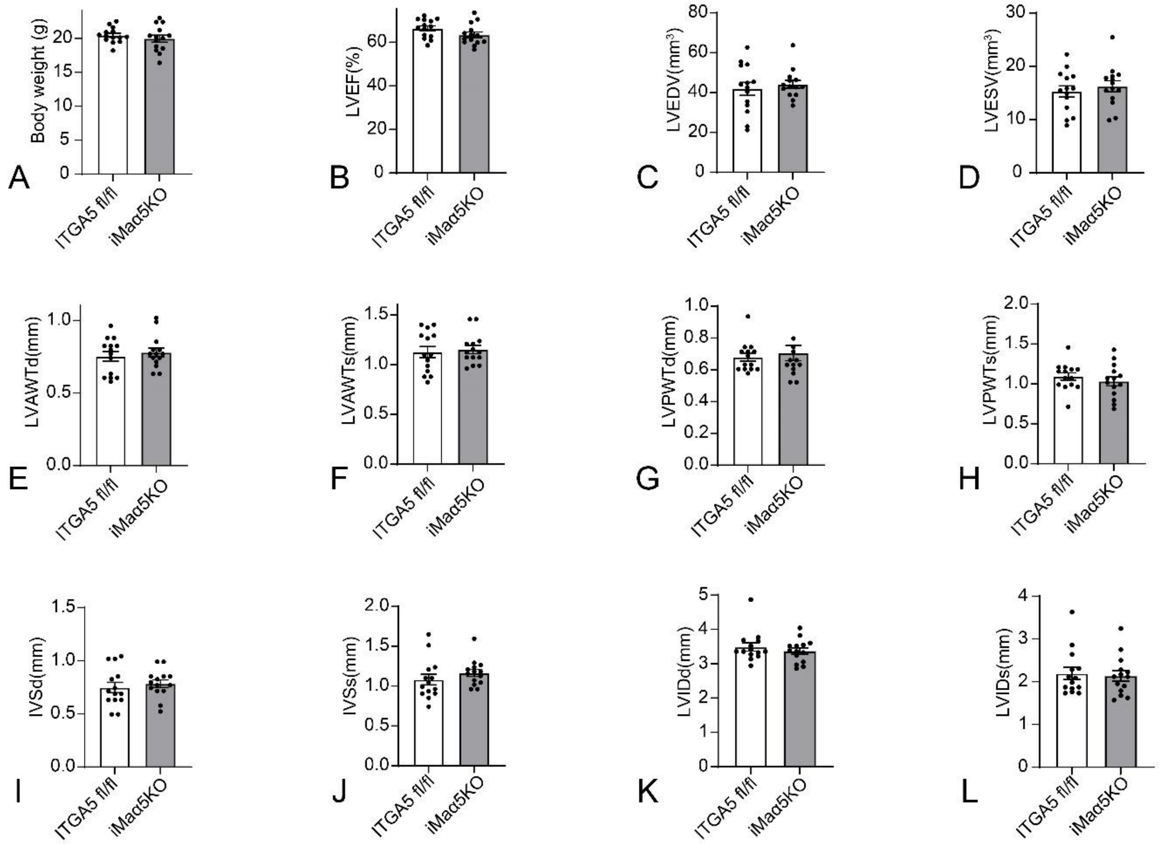

Supplemental Figure 14: Conditional macrophage-specific ITGA5 knockdown does not affect baseline body weight, cardiac dimensions and function. A: Young adult inducible macrophage-specific KO mice (iMaα5KO) and corresponding ITGA5 fl/fl animals had comparable body weight. B-L: Echocardiographic analysis showed that macrophage specific ITGA5 loss did not affect baseline cardiac dimensions and function. iMaα5KO mice and corresponding ITGA5 fl/fl animals had comparable left ventricular ejection fraction (LVEF; B), left ventricular end-diastolic volume (LVEDV; C), left ventricular end-systolic volume (LVESV; D), left ventricular anterior wall diastolic thickness (LVAWTd; E), left ventricular anterior wall systolic thickness (LVAWTs; F), left ventricular posterior wall diastolic thickness (LVPWTd; G), left ventricular posterior wall systolic thickness (LVPWTs; H), interventricular septal diastolic thickness (IVSd; I), interventricular septal systolic thickness(IVSs; J), left ventricular internal diastolic diameter (LVIDd; K), and left ventricular internal systolic diameter (LVIDs; L). (Data are shown as mean values +/- SEM, n=14 biologically independent experiments/group. Unpaired two-tailed Student's t test was used to compare the differences between the groups. Source data are provided as a Source Data file.

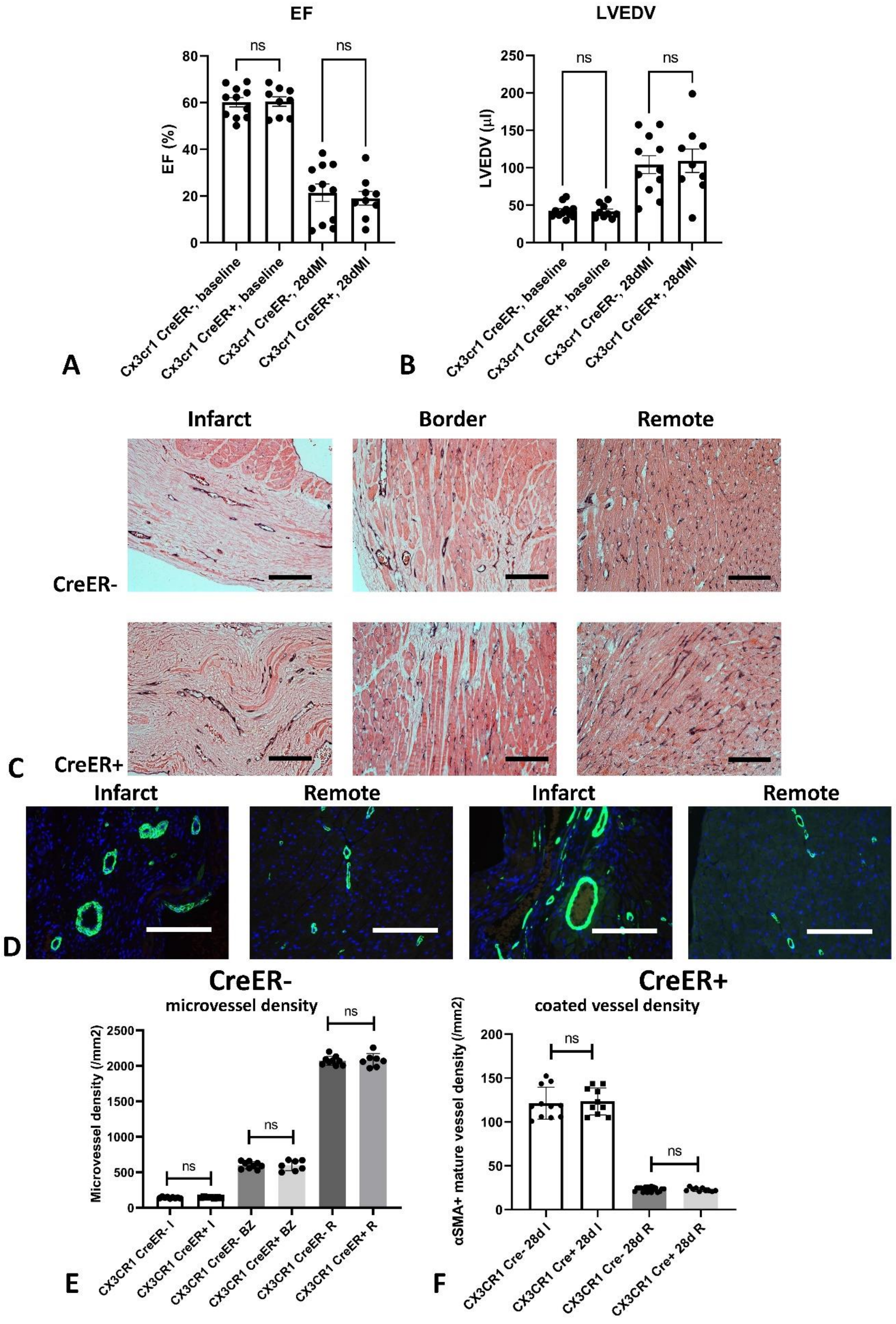
EF
ns
ns
80
60
40
20
0
EF (%)
Cx3cr1 CreER-, baseline
Cx3cr1 CreER+, baseline
Cx3cr1 CreER-, 28dMI
Cx3cr1 CreER+, 28dMI
A
LVEDV
ns
ns
250
200
150
100
50
0
LVEDV (μl)
Cx3cr1 CreER-, baseline
Cx3cr1 CreER+, baseline
Cx3cr1 CreER-, 28dMI
Cx3cr1 CreER+, 28dMI
B
Infarct
Border
Remote
CreER-
CreER+
C
Infarct
Remote
Infarct
Remote
D
CreER-
CreER+
microvessel density
ns
ns
ns
2500
2000
1500
1000
500
0
Microvessel density (/mm2)
CX3CR1 CreER- I
CX3CR1 CreER+ I
CX3CR1 CreER- BZ
CX3CR1 CreER+ BZ
CX3CR1 CreER- R
CX3CR1 CreER+ R
E
coated vessel density
ns
ns
200
150
100
50
0
αSMA+ mature vessel density (/mm2)
CX3CR1 Cre- 28d I
CX3CR1 Cre+ 28d I
CX3CR1 Cre- 28d R
CX3CR1 Cre+ 28d R
F

Supplemental Figure 15: Inducible Cre expression in CX3CR1^{CreER} mice has no impact on post-infarction systolic dysfunction, adverse remodeling and infarct angiogenesis. In order to exclude that the adverse remodeling and perturbed angiogenesis, noted in CX3CR1^{CreER};ITGA5 fl/fl mice 28 days after infarction, are due to tamoxifen-induced Cre expression (rather than ITGA5 loss), we compared function and infarct angiogenesis between tamoxifen-injected CX3CR1^{CreER} animals and corresponding Cre-negative controls undergoing 28 days coronary occlusion. Ejection fraction (EF, A) and LVEDV (B) were comparable between groups (CX3CR1CreER-, n=11 biologically independent experiments; CX3CR1CreER+, n=9 biologically independent experiments). Microvessel in the infarct zone, border zone and remote remodeling myocardium were labeled using CD31 immunohistochemistry (C), whereas the density of mature coated vessels (D) was assessed using α-SMA immunofluorescence (green). Quantitative analysis showed that Cre expression did not affect microvascular density (E) and the number of mature coated vessels (F) (For E: CX3CR1CreER-, n=9 biologically independent experiments; CX3CR1CreER+, n=7 biologically independent experiments; For F: CX3CR1CreER-, n=11 biologically independent experiments; CX3CR1CreER+, n=10 biologically independent experiments). Scalebar=100μm for panel C. Scalebar=140μm for panel D. Data are shown as mean values +/- SEM. Statistical comparisons between groups were performed using one-way ANOVA followed by the Sidak post-hoc test. Source data are provided as a Source Data file. NS: no significance

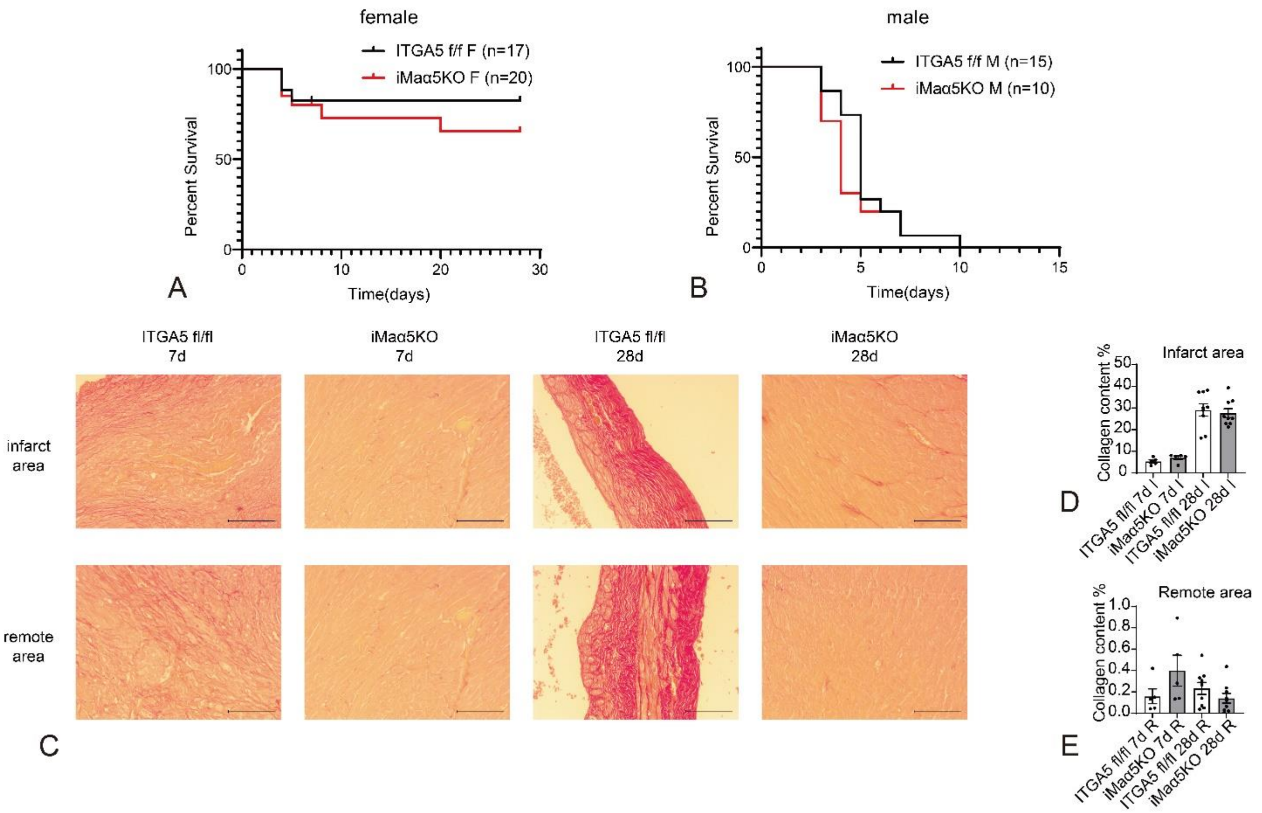

Supplemental Figure 16: Inducible macrophage-specific ITGA5 loss does not affect mortality, and collagen content after myocardial infarction. A-B: Comparison of survival curves between ITGA5 fl/fl and inducible macrophage–specific ITGA5 knockouts (iMaα5KO) after 7 to 28 days of permanent coronary occlusion showed no significant differences in female (A; ITGA5 fl/fl: n=17, iMaα5KO: n=20) and male mice (B; ITGA5 fl/fl: n=15, iMaα5KO: n=10). Please note the high mortality of male mice in comparison to female animals in both genotypes. Survival curves were compared using the log-rank test. C: Picrosirius red staining was used to label collagen fibers in the infarcted and in the remote remodeling myocardium of ITGA5 fl/fl and in iMaα5KO mice after 7-28 days of coronary occlusion. Quantitative analysis showed no significant effects of myeloid cell-specific ITGA5 loss on collagen content in the infarct zone and in the remote remodeling myocardium (D and E). Scalebar=100µm. (Data are shown as mean values +/- SEM. ITGA5 fl/fl: n=5 biologically independent experiments in the 7d group, n=9 biologically independent experiments in the 28d group; iMaα5KO: n=5 biologically independent experiments in 7d group, n=9 biologically independent experiments in 28d group). Statistical comparisons between groups were performed using one-way ANOVA, followed by Sidak post-hoc test. Source data are provided as a Source Data file.

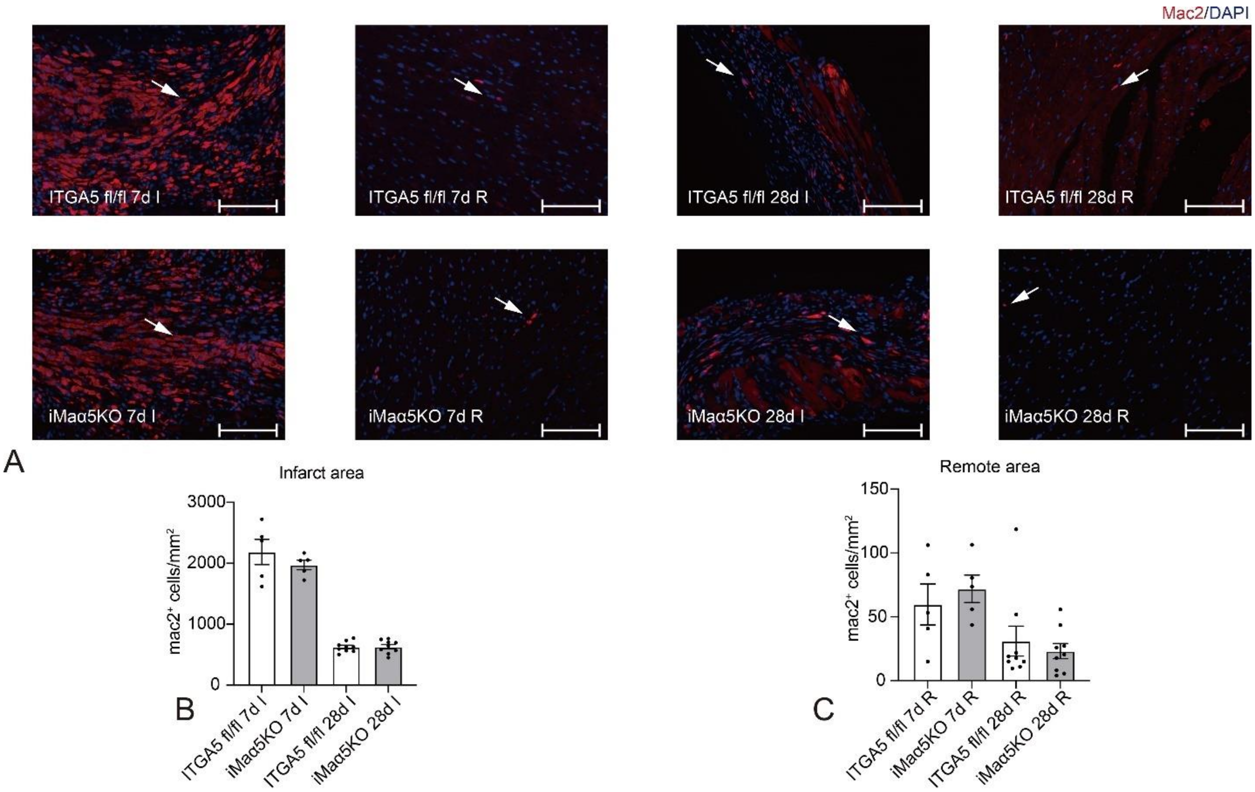

Supplemental Figure 17: Inducible macrophage-specific ITGA5 loss does not affect infiltration of the healing infarct with macrophages. A: Infarct macrophages were identified in ITGA5 fl/fl and in iMaα5KO infarcts(A) using Mac2 immunofluorescence (red fluorescence - arrows). B-C: Quantitative analysis showed that iMaα5KO mice and corresponding ITGA5 fl/fl animals had comparable macrophage density in the infarct and in the remote remodeling myocardium. Scalebar=100µm. (ITGA5 fl/fl: n=5 biologically independent experiments in the 7d group, n=9 biologically independent experiments in the 28d group; iMaα5KO: n=5 biologically independent experiments in the 7d group, n=9 biologically independent experiments in the 28d group). Data are shown as mean values +/- SEM. Statistical comparisons were performed using one-way ANOVA and post hoc Sidak test. Source data are provided as a Source Data file. I: infarct area; R: remote area.

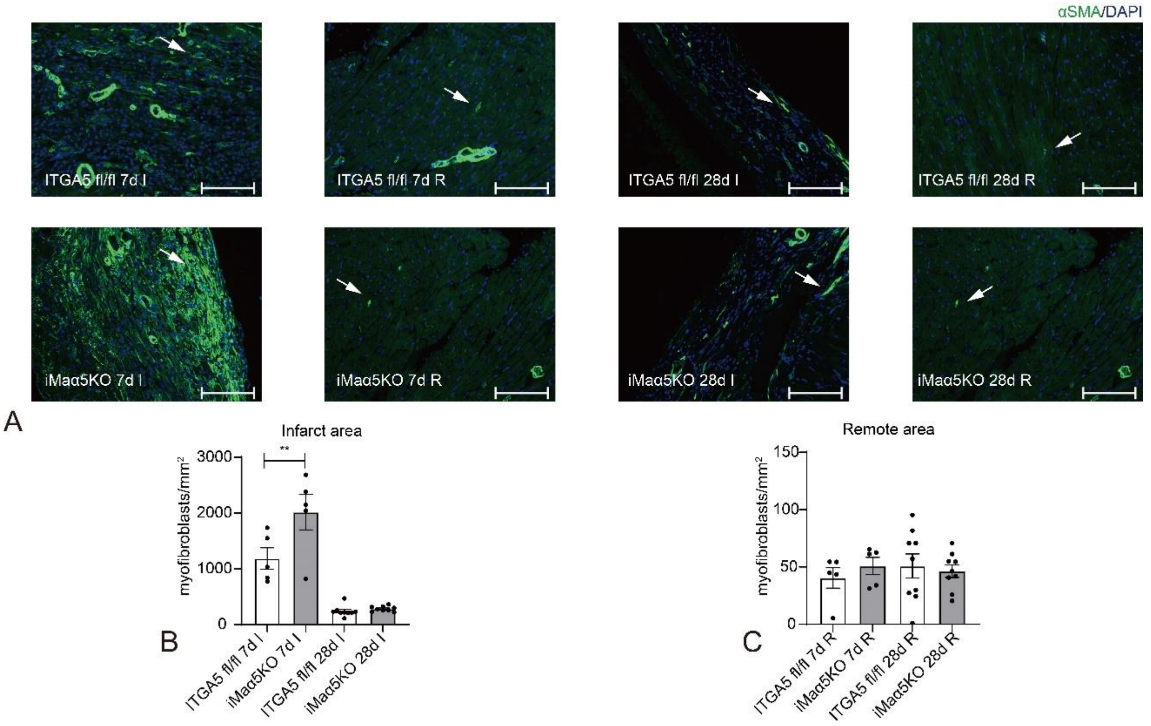

Supplemental Figure 18: Effects of inducible macrophage-specific ITGA5 loss on infarct myofibroblast density. Infarct myofibroblasts were identified in ITGA5 fl/fl and in iMaα5KO infarcts(A), as α-SMA-expressing cells located outside the vascular media (green fluorescence - arrows). Myofibroblast numbers in remote remodeling areas were low. B-C: Quantitative analysis showed that iMaα5KO mice had a transient increase in myofibroblast density at the 7-day timepoint, in comparison to ITGA5 fl/fl animals. However, no significant effects on myofibroblast density were noted at the 28-day timepoint. Myofibroblast density in the remote remodeling myocardium was comparable between groups. Scalebar=100μm. (**p<0.01, ITGA5 fl/fl: n=5 biologically independent experiments in the 7d group, n=9 biologically independent experiments in the 28d group; iMaα5KO: n=5 biologically independent experiments in the 7d group, n=9 biologically independent experiments in the 28d group). Data are shown as mean values +/- SEM. One-way ANOVA and post hoc Sidak test were used to compare differences between groups. Source data are provided as a Source Data file. I: infarct area; R: remote area.

	Log2FC	padj		Log2FC	padj		Log2FC	padj		Log2FC	padj
**RMp**			**Imp**			**PMp**			**Fib**		
Itga6	4.67	0	*Itgb7*	2.96	1.95E-302	Itga4	-0.73	1.1E-24	*Itga1*	7.88	0
Itga4	-1.45	0	*Itgal*	2.48	4.54E-237	Itgb1	0.82	1.3E-15	*Itgb1*	-0.85	4.6E-11
Itgb2	-1.13	0	*Itga6*	-3.72	1.59E-207	Itga5	1.27	2.3E-15			
Itgb5	4.18	0	*Itgam*	2.02	1.31E-153	Itga6	2.02	4.7E-15	**Mp11**		
Itgam	-0.93	4.93E-261	*Itgb2*	1.84	1.01E-134	Itgax	1.03	1.4E-13	*Itgal*	6.78	1E-99
Itgav	0.99	2.38E-94	*Itgb5*	-1.29	5.56E-77	Itgb2	0.51	1.9E-05	*Itga4*	3.94	1.1E-41
			Itgb1	-0.52	5.62E-67	Itgam	0.52	0.01	*Itgb1*	1.47	3.6E-18
**Mo**			*Itga4*	1.21	5.93E-58				*Itgb2*	2.6	3.4E-17
Itga4	4.47	0	*Itga6*	-0.66	1.85E-16	**Amp**			*Itgax*	2.61	2.6E-11
Itgal	7.42	0				*Itgax*	2.64	6E-143	*Itgb7*	2.02	1.4E-07
Itgb2	2.93	0	**Mp5**			*Itga5*	2.82	1E-142	*Itga9*	1.93	1.6E-06
Itgb7	3.86	0	*Itgam*	3.52	0	*Itga4*	-1.57	5E-88	*Itga6*	-2.02	1.1E-05
Itgb5	-4.85	0	*Itgb2*	2.19	0	*Itgb2*	2.02	1.5E-70	*Itgam*	2	1.7E-05
Itgam	1.31	1.72E-103	*Itgb5*	-2.2	5.28E-129	*Itgav*	2.12	1.6E-55	*Itgb5*	-0.71	2.7E-05
			Itga6	-2.02	4.94E-58	*Itga6*	2.4	2.3E-52	*Itgav*	2	0.0001
**CMp**						*Itgb1*	0.77	1.7E-16			
Itga9	2.09	1.64E-292	**Dc**			*Itgb5*	1.79	4.5E-16	**Mp12**		
Itgb5	-2.28	1.12E-223	Itgax	4.78	0				*Itgax*	4.98	1.3E-98
Itga6	-2.64	1.91E-219	Itgb7	5.85	0	**Gr**			*Itga4*	2.06	9E-18
Itgb2	-2.04	1.49E-204	Itgav	-2.06	6.22E-65	*Itgb1*	-5.09	8.4E-60	*Itgam*	2.06	5.5E-15
Itgb1	0.55	1.77E-195	Itgam	-1.29	1.48E-44	*Itgal*	2.03	2.2E-16	*Itgb2*	1.87	3E-12
Itgav	-1.45	2.06E-71	Itga4	1.03	5.29E-25				*Itgb1*	0.87	9.8E-07
Itgam	-1.23	3.07E-53	Itgb2	0.72	0.000000795						

Supplemental Figure 19: Cluster-specific patterns of integrin expression in CSF1R+ myeloid cells. Integrin genes upregulated or downregulated in specific clusters, in comparison with all other clusters are shown. The angiogenic macrophage cluster (Amp) and the proliferative macrophage cluster (PMp) had higher levels of *Itga5* expression, in comparison to all other clusters. Statistical comparisons were performed using the Wilcoxon rank sum test. Amp: angiogenic macrophages; PMp: proliferative macrophages; CMp: resident macrophages; RMp: reparative macrophages; Dc: dendritic cells; IMp: inflammatory macrophages; Mo: monocytes; Gr: granulocytes; Fib: fibroblasts; Mp: macrophages; padj: adjust p-value; FC: fold change. Source data are provided as a Source Data file. Raw scRNA-seq data were deposited in the NCBI’s Gene Expression Omnibus under accession number GSE227251.

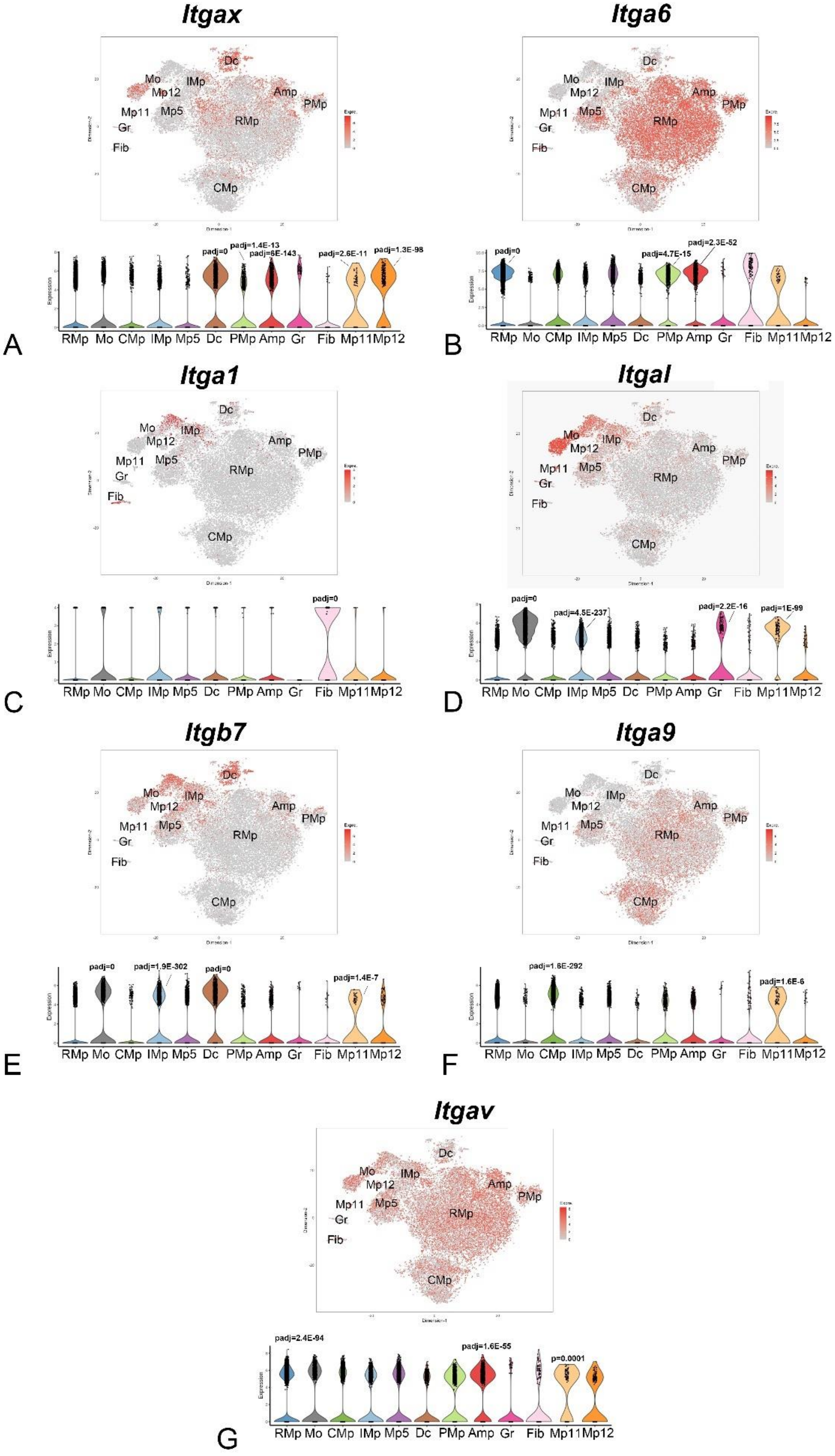
Itgax
Itga6
Itga1
Itgal
Itgb7
Itga9
Itgav
Dc
Mo
IMp
Mp12
Amp
PMp
Mp11
Mp5
RMp
Gr
Fib
CMp
padj=0
padj=1.4E-13
padj=6E-143
padj=2.6E-11
padj=1.3E-98
padj=4.7E-15
padj=2.3E-52
padj=4.5E-237
padj=2.2E-16
padj=1E-99
padj=1.9E-302
padj=1.4E-7
padj=1.6E-292
padj=1.6E-6
padj=2.4E-94
padj=1.6E-55
p=0.0001
RMp Mo CMp IMp Mp5 Dc PMp Amp Gr Fib Mp11 Mp12
A
B
C
D
E
F
G

Supplemental Figure 20: Cluster-specific patterns of integrin expression in infarct macrophages. A: The angiogenic macrophage cluster (Amp), proliferative macrophages (PMp), the minor resident macrophage clusters Mp11 and Mp12 and dendritic cells (Dc) exhibited higher levels of *Itgax* than other clusters, B: *Itga6* was expressed at higher levels by reparative macrophages (RMp), PMp and Amp. C: The small fibroblast cluster (Fib) had high expression of *Itga1*. D: Monocytes (Mo), granulocytes (Gr) and Mp11 cells exhibited high expression of *Itgal*. E: Itgb7 was highly expressed by monocytes, dendritic cells, inflammatory macrophages (IMp) and MP11 cells. F: CMp and Mp11 clusters had higher levels of *Itga9* than all other clusters. G: *Itgav* expression was higher in RMp, Amp and Mp11 clusters. Amp: angiogenic macrophages; PMp: proliferative macrophages; CMp: resident macrophages; RMp: reparative macrophages; Dc: dendritic cells; IMp: inflammatory macrophages; Mo: monocytes; Gr: granulocytes; Fib: fibroblasts; Mp: macrophages; padj: adjust p-value. Source data are provided as a Source Data file. Raw scRNA-seq data were deposited in the NCBI's Gene Expression Omnibus under accession number GSE227251.

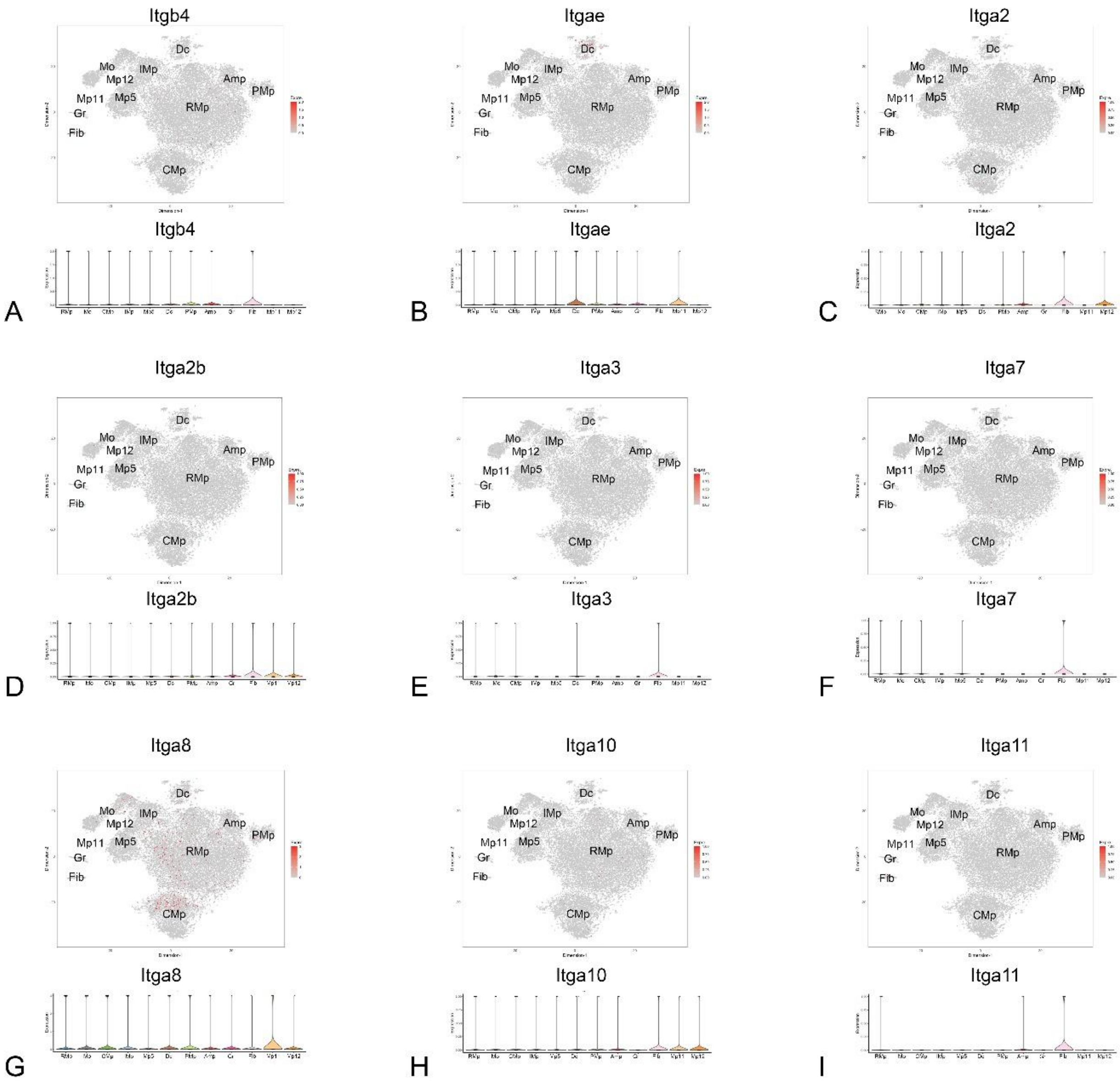

Supplemental Figure 21: Several integrin chains showed very low expression in cardiac macrophages. *Itgb4* (A), *Itgae* (B), *Itga2* (C), *Itga2b* (D), *Itga3* (E), *Itga7* (F), *Itga8* (G), *Itga10*, (H) and *Itga11* (I) expression was negligible in all macrophage clusters. Amp: angiogenic macrophages; PMp: proliferative macrophages; CMp: resident macrophages; RMp: reparative macrophages; Dc: dendritic cells; IMp: inflammatory macrophages; Mo: monocytes; Gr: granulocytes; Fib: fibroblasts; Mp: macrophages. Source data are provided as a Source Data file. Raw scRNA-seq data were deposited in the NCBI's Gene Expression Omnibus under accession number GSE227251.

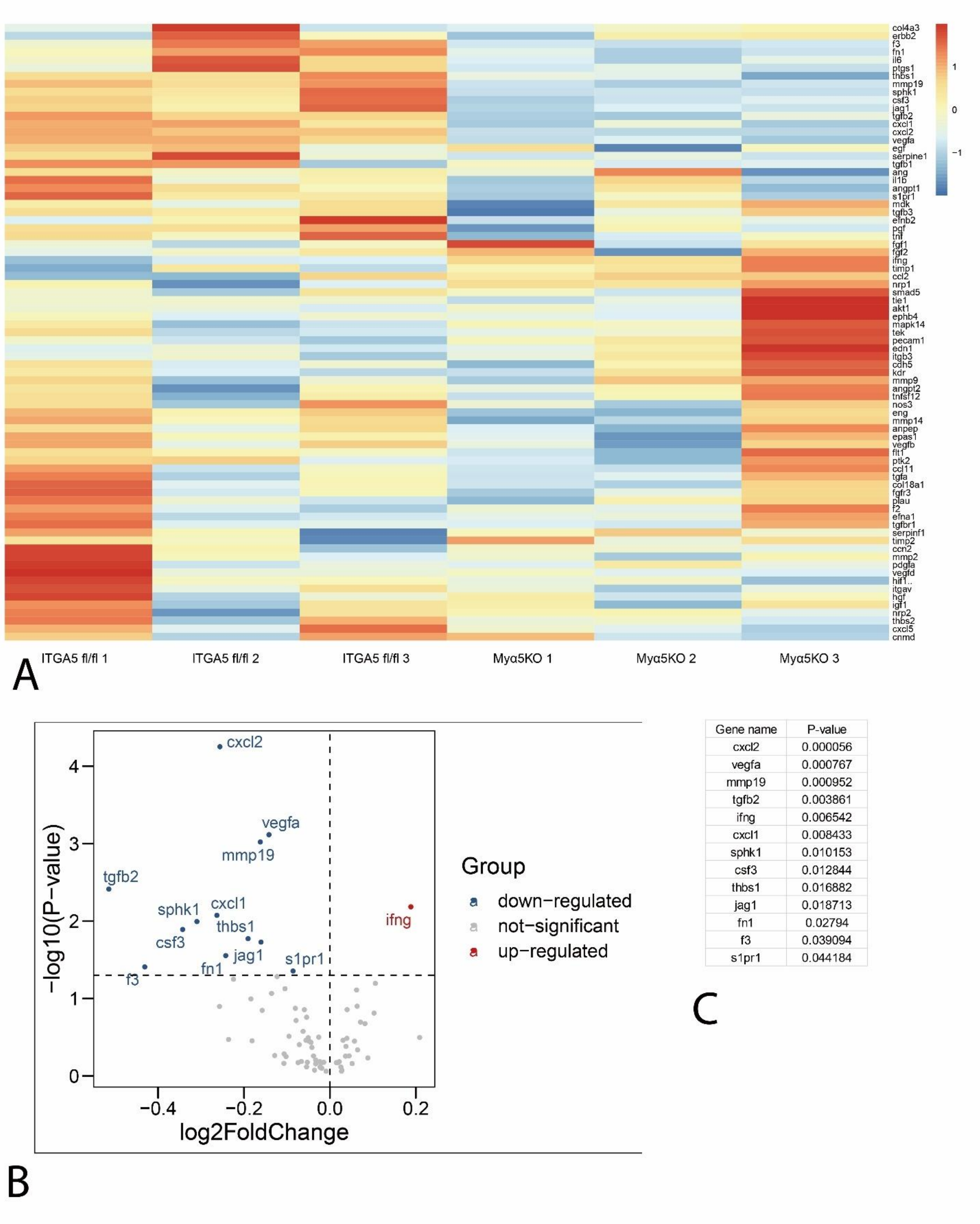

Gene name	P-value
cxcl2	0.000056
vegfa	0.000767
mmp19	0.000952
tgfb2	0.003861
ifng	0.006542
cxcl1	0.008433
sphk1	0.010153
csf3	0.012844
thbs1	0.016882
jag1	0.018713
fn1	0.02794
f3	0.039094
s1pr1	0.044184

Supplemental Figure 22: Effects of ITGA5 loss on synthesis of angiogenesis-related mediators by infarct macrophages. A - B: Heatmap (A) and volcano plot (B) summarizing PCR

array data, show relative expression of angiogenesis-associated genes in infarct macrophages (isolated as CD11b+/Ly6G- cells) from ITGA5 fl/fl and Myα5KO infarcts (after 7 days of coronary occlusion). In the heatmap, gene expression for each sample is represented by the z-score, as a measure of the distance from the mean of all samples. The scale in the heatmap represents the z score C: A list of angiogenesis-associated genes exhibiting statistically significant differences in expression between ITGA5 fl/fl and Myα5KO infarct macrophages. No adjustment for multiple comparisons was performed for statistical analysis of the PCR array data. It should be noted that the high variability between samples suggested by the heatmap reflects not only the inherent variability of in vivo experiments, but also the use of the z-score to illustrate gene expression, which accentuates the differences in low expression genes. (n=3 biologically independent experiments in each group). Unpaired two-tailed Student's t test was used to compare the differences between the groups. Source data are provided as a Source Data file.

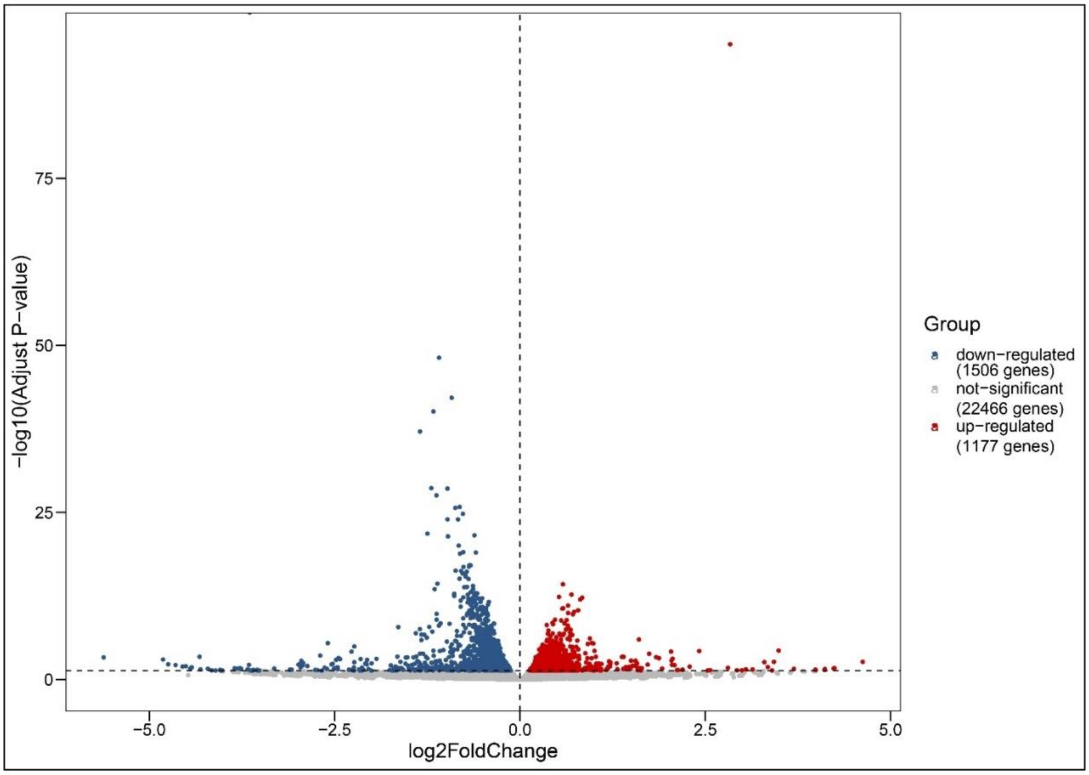

Supplemental Figure 23: Effects of ITGA5 loss on the transcriptomic profile of infarct macrophages. Volcano plot summarizing RNA-seq data on the differentially regulated genes between cardiac macrophages harvested from ITGA5 fl/fl and Myα5KO mice (7 days after coronary occlusion). In infarct macrophages, 1506 genes were downregulated and 1177 genes were upregulated upon ITGA5 loss. The RNA-seq processed data have been deposited in NCBI's Gene Expression Omnibus and are accessible through GEO SuperSeries accession number GSE190836.

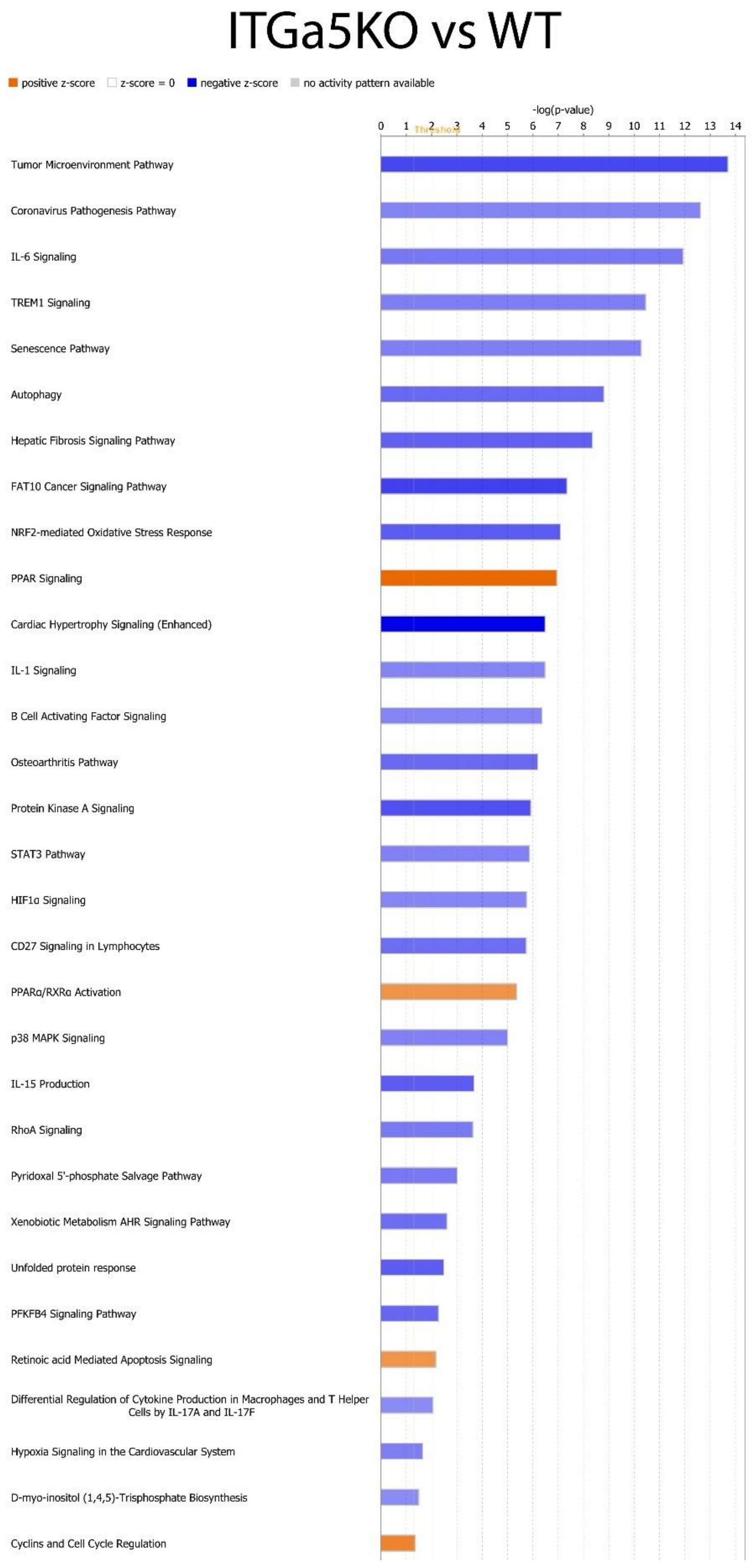

Supplemental Figure 24: Canonical pathways differentially modulated by ITGA5 loss in infarct macrophages. Ingenuity Pathway Analysis (IPA) of RNA-seq data identified a wide range

of canonical pathways, that were differentially modulated by ITGA5 loss in infarct macrophages. The RNA-seq processed data have been deposited in NCBI’s Gene Expression Omnibus and are accessible through GEO SuperSeries accession number GSE190836.

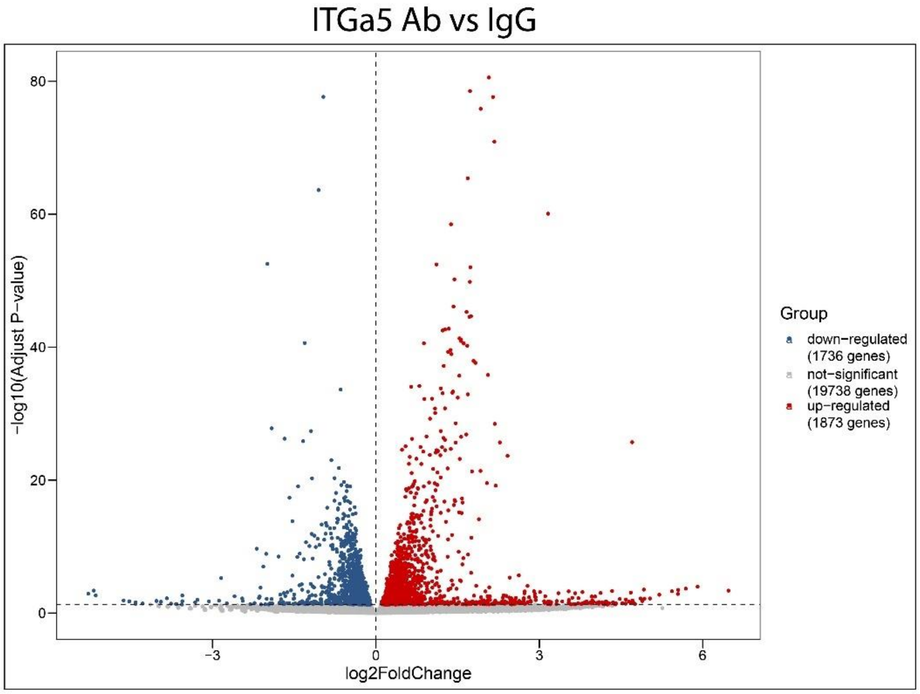

A

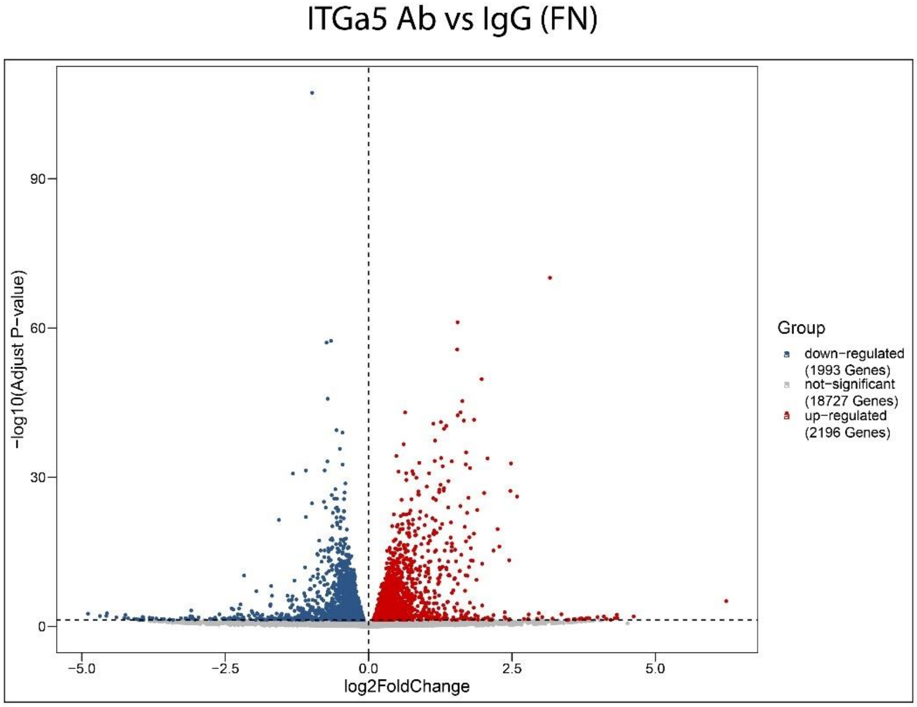

B

Supplemental Figure 25: Effects of ITGA5 blockade on the transcriptomic profile of bone marrow macrophages, in the presence or absence of fibronectin (FN). Volcano plots summarizing RNA-seq data on the differentially regulated genes in bone marrow macrophages

upon ITGA5 antibody neutralization blockade, in the absence (A) or presence (B) of fibronectin. ITGA5 blockade was associated with marked alterations in the macrophage transcriptome in the presence or absence of fibronectin (without fibronectin: 1736 downregulated genes and 1873 upregulated genes; with fibronectin: 1993 downregulated genes and 2196 upregulated genes). Ab: antibody; IgG: immunoglobulin G; FN: fibronectin. The RNA-seq data have been deposited in NCBI's Gene Expression Omnibus and are accessible through GEO SuperSeries accession number GSE190835.

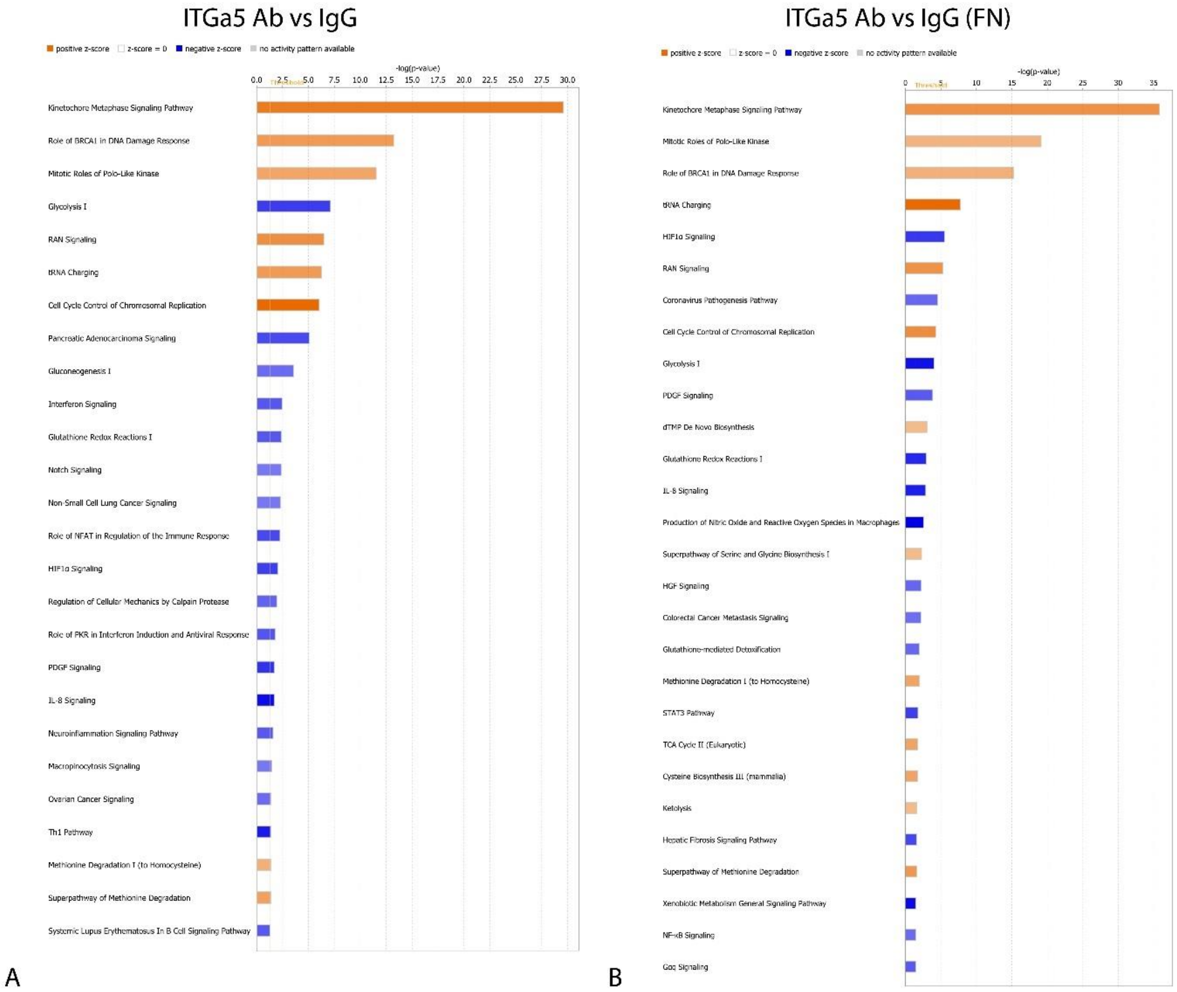

Supplemental Figure 26: Canonical pathways differentially modulated by ITGA5 blockade in bone marrow macrophages, in the presence or absence of fibronectin (FN). Ingenuity Pathway Analysis (IPA) identified numerous canonical pathways, that were differentially modulated by ITGA5 blockade, in the presence (B) or absence (A) of fibronectin. Ab: antibody; IgG: immunoglobulin G; FN: fibronectin. The RNA-seq data have been deposited in NCBI's Gene Expression Omnibus and are accessible through GEO SuperSeries accession number GSE190835.

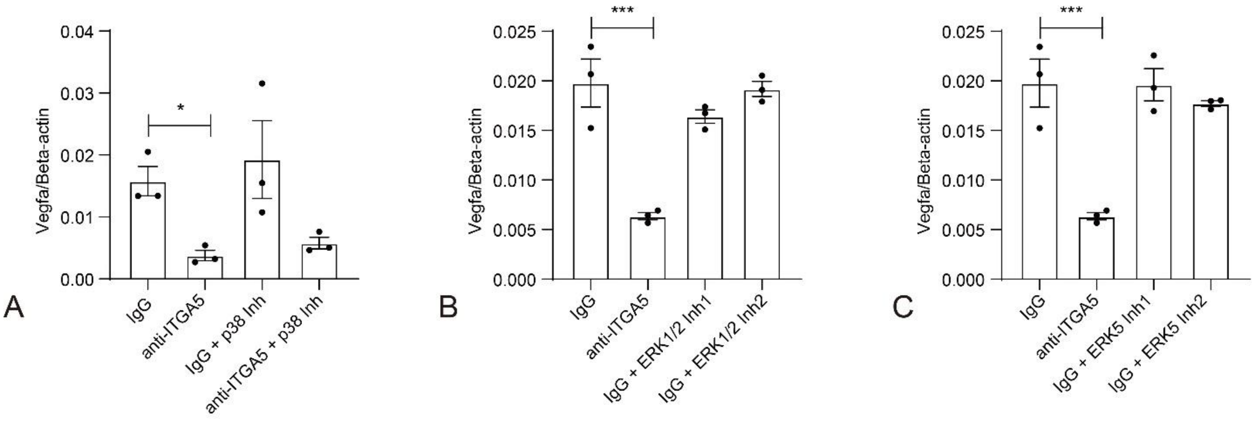

Supplemental Figure 27: ITGA5-mediated induction of the potent angiogenic mediator *Vegfa* does not involve p38 MAPK, Erk1/2 MAPK, or Erk5. ITGA5 blockade with a neutralizing antibody markedly attenuated *Vegfa* synthesis in bone marrow macrophages (*p<0.05, ***p<0.001, n=3/group), consistent with the critical role of ITGA5 in mediating expression of this angiogenic mediator. Treatment with the p38 inhibitor SB203580 (20µM), the Erk1/2 inhibitor U0126 (5µM), the Erk1/2 inhibitor PD98059 (50µM), the Erk5 inhibitor XMD8-92 (5µM), or the Erk5 inhibitor BIX02189 (5µM) for 24 hours did not reduce *Vegfa* synthesis in macrophages with intact ITGA5 signaling (IgG control group) in cells cultured in fibronectin-coated plates (A-C). Thus, p38, Erk1/2 and Erk5 do not play an important role in mediating ITGA5-induced *Vegfa* synthesis in macrophages. (Data are shown as mean values +/- SEM. *p<0.05, ***p<0.001, n=3 biologically independent experiments for each group). One-way ANOVA and the Sidak post-hoc test were used to compare differences between groups. Source data are provided as a Source Data file. IgG: immunoglobulin G.

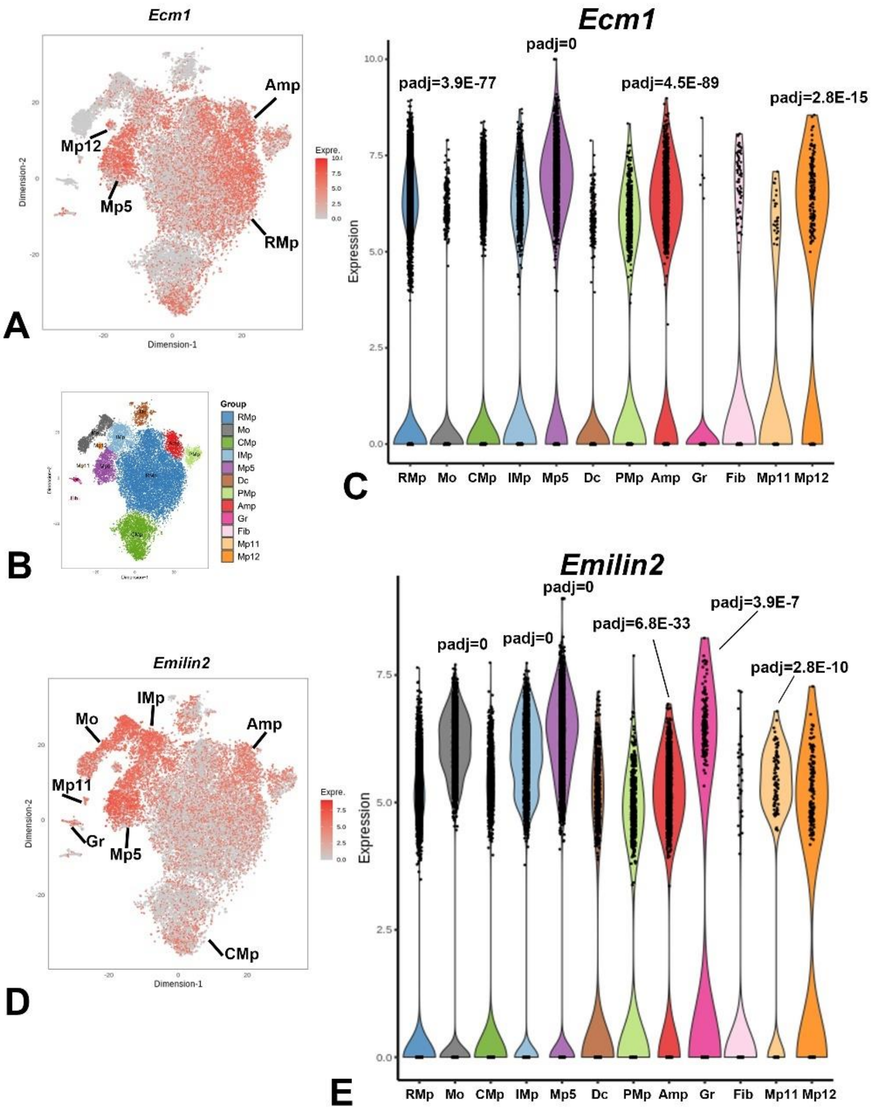

Supplemental Figure 28: Cluster-specific patterns of Emilin2 and Ecm1 expression in CSF1R+ myeloid cells. ScRNA-seq showed that both *Ecm1* and *Emilin2* were broadly expressed by CSF1R+ macrophage clusters. A, C: Angiogenic macrophages (Amp), reparative macrophages (RMp), and the Mp5 and Mp12 clusters had higher expression of *Ecm1* than other clusters. D-E: Angiogenic macrophages (Amp), monocytes (Mo), granulocytes (Gr), and the Imp, CMp, Mp5 and Mp11 clusters had higher expression of *Emilin2*. Panel B illustrates the color coding for cluster identification in violin plots. Amp: angiogenic macrophages; PMp: proliferative macrophages; CMp: resident macrophages; RMp: reparative macrophages; Dc: dendritic cells; IMp: inflammatory macrophages; Mo: monocytes; Gr: granulocytes; Fib: fibroblasts; Mp: macrophages; padj: adjusted p-value. Source data are provided as a Source Data file. Raw scRNA-seq data were deposited in the NCBI’s Gene Expression Omnibus under accession number GSE227251.

SUPPLEMENTAL TABLES:

Supplemental Table 1: Summary metrics of scRNA-seq data

Sample	Control	Infarct1	Infarct2	Infarct3
Number of Mice	3	1	1	1
Estimated Number of Cells	10,314	10,424	10,804	10,144
Mean Reads per Cell	27,239	24,795	24,999	25,723
Median Genes per Cell	2,277	2,569	2,491	2,558
Number of Reads	280,946,984	258,458,270	270,092,399	260,938,264
Valid Barcodes	98.1%	98.1%	98.2%	98.1%
Sequencing Saturation	40.8%	39.7%	41.8%	41.2%
Fraction Reads in Cells	82.6%	91.6%	92.2%	87.6%
Total Genes Detected	19,099	18,714	18,653	18,879
Median UMI Counts per Cell	6,368	8,547	8,470	8,492
Number of Cells after filtering	8,751	9,222	9,632	8,330

UMI: unique Molecular Identifier

Supplemental Table 2: Clusters of CSF1R+ cells in control and infarcted hearts.

Cluster	Control (%)	Inf1 (%)	Inf2 (%)	Inf3 (%)
RMp	5.530796	54.48926	75.66445	69.86795
Mo	21.49469	1.875949	2.159468	3.253301
CMp	56.94206	1.236174	1.142027	1.044418
IMp	2.411153	12.07981	6.478405	4.273709
Mp5	0.857045	16.14617	1.484635	8.47539
Dc	3.188207	5.617003	3.42608	3.553421
PMp	1.519826	2.374756	3.47799	3.32533
Amp	1.416981	4.836261	5.346761	4.57383
Gr	0.822763	0.769898	0.633306	1.332533
Fib	2.216889	0.466276	0.155731	0.240096
Mp11	1.177008	0.010844	0	0
Mp12	2.42258	0.097593	0.031146	0.060024

Amp: angiogenic macrophages; PMp: proliferative macrophages; CMp: resident macrophages; RMp: reparative macrophages; Dc: dendritic cells; IMp: inflammatory macrophages; Mo: monocytes; Gr: granulocytes; Fib: fibroblasts; Mp: macrophages; Inf: infarct.

Supplemental Table 3: Top 20 upstream regulators predicted to be inhibited in ITGA5 KO infarct macrophages (ranked by z-score)

Upstream Regulator	Predicted Activation State	Activation z-score	p-value of overlap
STAT3	Inhibited	-5.913	2.09E-42
IL4	Inhibited	-5.731	2.43E-50
TGFB1	Inhibited	-5.715	2E-49
TNF	Inhibited	-5.483	3.57E-65
HIF1A	Inhibited	-5.355	3.5E-24
NFE2L2	Inhibited	-5.119	2.6E-15
TNFSF11	Inhibited	-5.028	1.43E-38
TRIM24	Inhibited	-4.969	4.08E-25
PNPT1	Inhibited	-4.889	7.15E-27
IL1A	Inhibited	-4.773	8.74E-23
IL1B	Inhibited	-4.524	3.6E-45
NFAT5	Inhibited	-4.512	7.16E-23
ATF4	Inhibited	-4.375	1.74E-14
PTGER4	Inhibited	-4.313	8.54E-22
RELA	Inhibited	-4.27	5.1E-27
IL33	Inhibited	-4.249	1.83E-38
ANGPT2	Inhibited	-4.23	7.79E-09
IL6	Inhibited	-4.23	7.47E-34
PGR	Inhibited	-4.212	2.53E-21
AGT	Inhibited	-4.186	2.59E-22

Supplemental Table 4: Top 20 upstream regulators predicted to be activated in ITGA5 KO infarct macrophages (ranked by z-score)

Upstream Regulator	Predicted Activation State	Activation z-score	p-value of overlap
IRF7	Activated	5.008	4.94E-29
IRF3	Activated	4.733	3.4E-29
STEAP3	Activated	3.729	0.000000374
IFNB1	Activated	3.621	3.78E-32
PML	Activated	3.245	1.43E-16
IFNL1	Activated	3.216	3.47E-15
IFNA2	Activated	3.127	4.62E-23
STAT1	Activated	3.056	6.44E-39
DUSP1	Activated	2.953	2.43E-17
GFI1	Activated	2.944	1.72E-20
TAF4	Activated	2.909	0.000000757
SMAD7	Activated	2.81	2.16E-09
PARP9	Activated	2.789	0.000000941
PTPN11	Activated	2.783	0.00000122
SIGIRR	Activated	2.775	0.00000383
CAT	Activated	2.763	0.0000633
NR0B2	Activated	2.76	0.124
PDPK1	Activated	2.701	0.00000354
SFTPA1	Activated	2.635	6.39E-09
ATG16L1	Activated	2.611	0.000000436

Supplemental Table 5: Top 20 regulators predicted to be inhibited upon ITGA5 blockade in vitro (ranked by z-score)

Upstream Regulator	Predicted Activation State	Activation z-score	p-value of overlap
NUPR1	Inhibited	-7.036	3.98E-28
TP53	Inhibited	-6.297	2.57E-61
IRF7	Inhibited	-5.666	1.62E-11
CDKN2A	Inhibited	-5.651	1.4E-21
IFNG	Inhibited	-5.202	5.34E-22
TGM2	Inhibited	-5.05	5.62E-10
IRF3	Inhibited	-4.967	5.84E-09
IFNA2	Inhibited	-4.637	5.85E-09
STAT1	Inhibited	-4.448	1.41E-13
SMARCB1	Inhibited	-4.395	1.62E-11
HIF1A	Inhibited	-4.354	4.73E-10
PRL	Inhibited	-4.313	9.7E-09
STAT4	Inhibited	-4.156	6.31E-06
IFNL1	Inhibited	-4.121	1.15E-07
IKBKG	Inhibited	-4.075	0.00345
CDKN1A	Inhibited	-4.035	9.97E-35
FOXO3	Inhibited	-3.963	1.17E-19
TP73	Inhibited	-3.932	8.55E-12
TCF3	Inhibited	-3.883	1.09E-20
ETS2	Inhibited	-3.804	6.27E-05

Supplemental Table 6: Top 20 regulators predicted to be activated upon ITGA5 blockade in vitro (ranked by z-score)

Upstream Regulator	Predicted Activation State	Activation z-score	p-value of overlap
TBX2	Activated	5.127	3.64E-16
PNPT1	Activated	4.876	1.02E-15
MYC	Activated	4.797	2.01E-26
ESR2	Activated	4.73	5.37E-10
KDM1A	Activated	4.394	0.00246
E2F3	Activated	4.148	2.04E-10
FOXM1	Activated	4.079	1.12E-16
NKX2-3	Activated	3.896	3.95E-06
S100A6	Activated	3.873	5.59E-09
TRIM24	Activated	3.811	2.31E-10
ACKR2	Activated	3.771	1.21E-08
VDR	Activated	3.738	1.87E-08
RARA	Activated	3.426	0.00243
CASR	Activated	3.388	1.75E-11
TXNRD1	Activated	3.359	4.41E-06
MYBL2	Activated	3.35	9.32E-08
AREG	Activated	3.256	2.74E-12
CSF2	Activated	3.225	2.28E-30
GSR	Activated	3.207	1.5E-06
MYCL	Activated	3.205	0.000076

Supplemental Table 7: Top 20 regulators predicted to be inhibited upon ITGA5 blockade in fibronectin-stimulated macrophages (ranked by z-score)

Upstream Regulator	Predicted Activation State	Activation z-score	p-value of overlap
**NUPR1**	Inhibited	-7.628	1.23E-22
**CDKN2A**	Inhibited	-6.756	1.28E-20
**TP53**	Inhibited	-6.741	4.65E-68
**KDM5B**	Inhibited	-4.268	0.000178
**TGM2**	Inhibited	-4.201	0.00000626
**CDKN1A**	Inhibited	-4.084	1.12E-24
**HNF4A**	Inhibited	-4.084	8.55E-19
**ETS2**	Inhibited	-3.944	0.000808
**CEBPA**	Inhibited	-3.903	0.0000432
**SMARCB1**	Inhibited	-3.902	0.000000452
**PTTG1**	Inhibited	-3.879	0.0000186
**MXD1**	Inhibited	-3.811	8.9E-09
**ESRRG**	Inhibited	-3.757	0.0000299
**STAT4**	Inhibited	-3.749	0.000151
**TET2**	Inhibited	-3.665	0.000407
**HIF1A**	Inhibited	-3.644	5.97E-10
**TP73**	Inhibited	-3.641	1.66E-10
**GATA1**	Inhibited	-3.518	0.00000263
**FOXO3**	Inhibited	-3.494	1.57E-19
**PTGER4**	Inhibited	-3.479	0.0000726

Supplemental Table 8: Top 20 regulators predicted to be activated upon ITGA5 blockade in fibronectin-stimulated macrophages (ranked by z-score)

Upstream Regulator	Predicted Activation State	Activation z-score	p-value of overlap
MYC	Activated	6.569	1.29E-37
TBX2	Activated	5.496	9.66E-16
ESR2	Activated	4.559	4.79E-12
MYCL	Activated	4.549	0.000000624
TXNRD1	Activated	3.769	0.00000153
E2F2	Activated	3.679	0.00035
GSR	Activated	3.638	0.000000247
AREG	Activated	3.589	0.000000296
E2F3	Activated	3.563	9.93E-10
FOXM1	Activated	3.539	4.7E-17
RARA	Activated	3.507	0.0998
S100A6	Activated	3.5	3.34E-08
CDK19	Activated	3.414	0.00000087
MYBL2	Activated	3.35	0.00000143
PTGER2	Activated	3.25	8.75E-20
CSF2	Activated	3.174	3.64E-31
KDM1A	Activated	3.05	0.1
MLXIPL	Activated	3.041	0.0609
VDR	Activated	3.024	0.00000195
ASXL1	Activated	3	0.0271

Supplemental Table 9: Intracellular pathways predicted to be modulated by ITGA5 in both infarct macrophages and in cultured bone marrow macrophages.

Pathway	In vivo		In vitro	
	z-score	P value	z-score	p-value
**Nrf2**	-2.83	8.09*10-8	-1.4	3.97*10-6
**PI-3K/Akt**	-1.78	9.22*10-7	-1.8	3.46*10-3
**Erk5**	-1.6	5.5*10-6	-0.5	4.1*10-3
**VEGF signaling**	-1.39	2.15*10-4	-1.8	6.3*10-3
**HIF1a**	-2.12	1.78*10-6	-3.02	8.68*10-3
**MAPK signaling**	-0.5	6.63*10-8	-1.13	1.27*10-2
**P38 signaling**	-2.18	9.97*10-6	-1.0	1.68*10-2

Supplemental Table 10: Effects of ITGA5 loss on levels of macrophage-derived secreted mediators in vivo and in vitro

Gene	In vivo (ITGA5 KO vs C)		In vitro ITGA5 Ab vs IgG		In vitro ITGA5 Ab vs IgG (+FN)	
	Log2FC	padj	Log2FC	padj	Log2FC	padj
Il1a	-1.32982	1.15E-05	0.26289974	0.82676888	-0.27096	0.769378
Il1b	-0.29329	0.001638	0.98569339	0.37084835	0.230047	1
Il6	-0.9569	0.008423	-0.1210481	1	1.483082	1
Il10	-1.12678	3.68E-25	0.21844332	1	0.912846	1
Nrg1	-1.11391	1.66E-12	2.43062407	1	-	-
Tgm2	-0.60355	1.95E-09	-0.0620842	0.6778824	-0.15384	0.013595
Adamts9	-0.80036	2.11E-09	-	-	-	-
Serpine1	-0.65486	7.60E-09	-0.3374535	0.42280972	-0.52118	0.05066
CSF3	-1.09347	7.42E-07	1.62280608	1	1.98124	1
Cxcl3	-1.64131	1.19E-06	-0.3337757	0.67510957	-0.23908	0.690111
Emilin2	-0.45382	6.39E-06	-0.3142762	3.51E-05	-0.28371	1.60E-08
Tgfb3	-0.50066	6.88E-05	0.50788626	0.37084835	0.494066	0.266027
Ecm1	-0.39054	0.000104	-0.440549	9.41E-05	-0.33252	2.46E-05
Hbegf	-0.56118	0.00012	-0.2051027	0.6054942	0.155299	0.646756
Lox	-0.62318	0.000294	-0.549416	1	0.178629	1
Loxl1	-0.49034	0.003543	0.57834357	1	-0.2024	1
Areg	-0.68249	0.000358	-	-	0.133781	1
Pdgfa	-0.36713	0.001376	-0.2078066	0.31780296	-0.35518	0.014723
Fbln2	-0.30581	0.012128	-0.5318643	1	-0.96132	1
Cxcl5	-1.34796	0.003062	0.83610292	1	-1.02087	1

FN: fibronectin; padj: adjusted p-value; FC: fold change.

Supplemental Table 11: Antibodies used for immunofluorescence and immunohistochemical staining.

**Antibodies**	**Catalog Number**	**Clone**	**Dilution**
α-SMA	Sigma, F3777	1A4	1:150
CD31	Cell Signaling Technology, 77699	D8V9E	1:100
ITGA5	Abcam, ab150361	EPR7854	1:100
FITC Anti-GFP	Abcam, ab6662	N/A	1:100
Mac-2	Cedarlane, CL8942AP	M3/38	1:200
Alexa Fluor 488 donkey anti-rabbit IgG (H+L)	Thermofisher, A21206	N/A	1:500
Alexa Fluor 594 donkey anti-rabbit IgG (H+L)	Thermofisher, A21207	N/A	1:500
Alexa Fluor 594 donkey anti-rat IgG (H+L)	Thermofisher, A21209	N/A	1:500

Supplemental Table 12: Antibodies used for in vitro neutralization experiments

**Antibodies**	**Catalog Number**	**Clone**	**Dilution**
ITGA5	Biolegend, 103910	HMα5-1	1:100
IgG	Biolegend, 400902	HTK888	1:50

Supplemental Table 13: Antibodies for flow cytometric analysis of ITGA5/CD49e expression

**Antibody**	**Catalog Number**	**Clone**	**Dilution**
CD45-PE-Cy5	BD Pharmingen, 553082	30-F11	1:1000
CD11b-APC/Cyanine7	Biolegend, 101226	M1/70	1:1000
Ly6G-PerCP/Cyanine5.5	Biolegend, 127616	1A8	1:1000
MerTK-PE	Biolegend, 151506	2B10C42	1:100
CD64-FITC	Biolegend, 139316	X54-5/7.1	1:100
CD49e-APC	Biolegend, 103814	5H10-27	1:100
Rat IgG2a, κ Isotype Control-APC	Biolegend, 400512	RTK2758	1:100
CD16/32	BD Pharmingen, 553142	2.4G2	1:100

Supplemental Table 14: Antibodies for flow cytometric analysis of immune cells in myeloid cell-specific ITGA5 KO and control mice

Antibody	Catalog Number	Clone	Dilution
CD45-PE-Cy5	BD Pharmingen, 553082	30-F11	1:1000
CD11b-APC/Cyanine7	Biolegend, 101226	M1/70	1:1000
Ly6G-PerCP/Cyanine5.5	Biolegend, 127616	1A8	1:1000
MerTK-PE	Biolegend, 151506	2B10C42	1:100
CD64-FITC	Biolegend, 139316	X54-5/7.1	1:100
CD3e-APC	Biolegend, 100311	145-2C11	1:100
CD16/32	BD Pharmingen, 553142	2.4G2	1:100

Supplemental Table 15: Antibodies used for western blotting

Antibodies	Catalog Number	Clone	Dilution
ITGA5	Abcam, ab150361	EPR7854	1:1000
p-AKT	Cell Signaling Technology, 4060	D9E	1:1000
AKT	Cell Signaling Technology, 9272	Polyclonal	1:1000
p-P38	Cell Signaling Technology, 4511	D3F9	1:1000
P38	Cell Signaling Technology, 8690	D13E1	1:1000
p-ERK1/2	Cell Signaling Technology, 4370	D13.14.4E	1:1000
ERK1/2	Cell Signaling Technology, 4695	137F5	1:1000
p-FAK	Cell Signaling Technology, 3283	Polyclonal	1:1000
FAK	Cell Signaling Technology, 3285	Polyclonal	1:1000
beta Actin	Cell Signaling Technology, 4970	13E5	1:1000
Anti-rabbit IgG, HRP-linked Antibody	Cell Signaling Technology, 7074	N/A	1:2000

Protective effects of macrophage-specific integrin α5 in myocardial infarction are associated with accentuated angiogenesis

Ruoshui Li, Bijun Chen, Akihiko Kubota, Anis Hanna, Claudio Humeres, Silvia C Hernandez, Yang Liu, Richard Ma, Izabela Tuleta, Shuaibo Huang, Harikrishnan Venugopal, Fenglan Zhu, Kai Su, Jun Li, Jinghang Zhang, Deyou Zheng, and Nikolaos G Frangogiannis.

Full Unedited/Uncropped blots for Supplemental Figures

Full unedited gel for Supplemental Figure 5

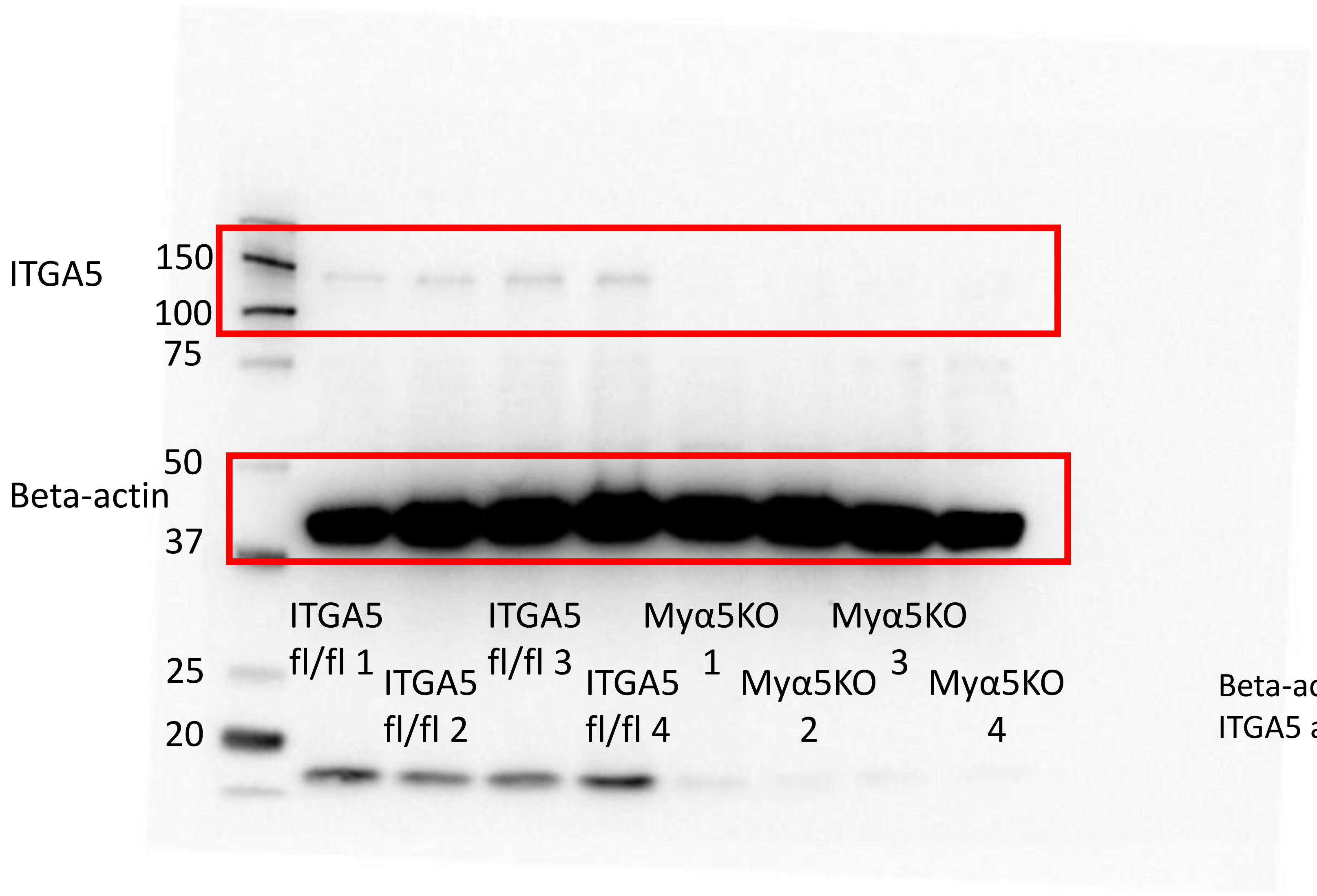

Beta-actin antibody (Cell Signaling Technology, #4970)
ITGA5 antibody (Cell Signaling Technology, #150361)
