# Supplementary material for: Protective effects of macrophage-specific integrin α5 in myocardial infarction are associated with accentuated angiogenesis
Source: Nat Commun. 2023 Nov 20;14:7555. doi: 10.1038/s41467-023-43369-x (PMC10662477; doi:10.1038/s41467-023-43369-x)
Supplement: Supplementary file 3 — Reporting Summary [file 41467_2023_43369_MOESM3_ESM.pdf]

nature portfolio

Corresponding author(s): Nikolaos G Frangogiannis

Last updated by author(s): Oct 28, 2023

Reporting Summary

Nature Portfolio wishes to improve the reproducibility of the work that we publish. This form provides structure for consistency and transparency in reporting. For further information on Nature Portfolio policies, see our Editorial Policies and the Editorial Policy Checklist.

Statistics

For all statistical analyses, confirm that the following items are present in the figure legend, table legend, main text, or Methods section.

n/a	Confirmed	
☐	☒	The exact sample size (*n*) for each experimental group/condition, given as a discrete number and unit of measurement
☐	☒	A statement on whether measurements were taken from distinct samples or whether the same sample was measured repeatedly
☐	☒	The statistical test(s) used AND whether they are one- or two-sided<br>*Only common tests should be described solely by name; describe more complex techniques in the Methods section.*
☐	☒	A description of all covariates tested
☐	☒	A description of any assumptions or corrections, such as tests of normality and adjustment for multiple comparisons
☐	☒	A full description of the statistical parameters including central tendency (e.g. means) or other basic estimates (e.g. regression coefficient) AND variation (e.g. standard deviation) or associated estimates of uncertainty (e.g. confidence intervals)
☐	☒	For null hypothesis testing, the test statistic (e.g. *F*, *t*, *r*) with confidence intervals, effect sizes, degrees of freedom and *P* value noted<br>*Give P values as exact values whenever suitable.*
☒	☐	For Bayesian analysis, information on the choice of priors and Markov chain Monte Carlo settings
☒	☐	For hierarchical and complex designs, identification of the appropriate level for tests and full reporting of outcomes
☒	☐	Estimates of effect sizes (e.g. Cohen's *d*, Pearson's *r*), indicating how they were calculated

Our web collection on statistics for biologists contains articles on many of the points above.

Software and code

Policy information about availability of computer code

Data collection	Zen v2.6 Pro software (Carl Zeiss Microscopy) was used for obtaining light and fluorescence microscopy images. Vevo LAB v5.6.1 (VisualSonics) was used to obtain echocardiographic data.
Data analysis	Zen 2.6 Pro software (Carl Zeiss Microscopy) was used for histologic analysis, and for machine learning based quantifications of immunostaining. TopHat2 algorithm alignment program v.2.0.9 (Computational Biology Dept. at Johns Hopkins Uni.) for mapping of RNAseq reads. Scripture (beta2) and Cufflinks v.2.1.1 algorithm (Center for Bioinformatics and Computational Biology at the University of Maryland, College Park) for assembly of RNAseq mapped reads. HTSeq software v.0.6.1 (the European Molecular Biology Laboratory) was used to count the number of reads mapped to each gene. DESeq2 R software package v.1.10.1 (the European Molecular Biology Laboratory) was used for gene differential expression analysis. RStudio software (v.1.4.1717) was used for data visualization. Ingenuity pathway analysis (Qiagen) was used for RNAseq enrichment analysis and identification of upstream regulators. Image Lab v.3.0 software (Bio-Rad Laboratories) was used for western blot band densitometry analysis. GraphPad Prism v.8 (GraphPad Software, Inc.) was used for statistical analysis and data visualization. FlowJo software (BD Biosciences) was used for FACS analysis.<br>For scRNA-seq:<br>Cell Ranger v3.1.0, from 10x Genomics, was used to align scRNA-seq reads to the mouse reference genome (assembly and annotation, mm10-3.0.0).<br>RISC v1.0 was used for scRNA-seq data integration (Liu et al., PMID: 33767393).

For manuscripts utilizing custom algorithms or software that are central to the research but not yet described in published literature, software must be made available to editors and reviewers. We strongly encourage code deposition in a community repository (e.g. GitHub). See the Nature Portfolio guidelines for submitting code & software for further information.

Data

Policy information about availability of data

All manuscripts must include a data availability statement. This statement should provide the following information, where applicable:

- Accession codes, unique identifiers, or web links for publicly available datasets
- A description of any restrictions on data availability
- For clinical datasets or third party data, please ensure that the statement adheres to our policy

All RNA-seq processed data have been deposited in NCBI's Gene Expression Omnibus and are accessible through GEO SuperSeries accession number GSE190837 (GSE190835 in vitro study and GSE190836 in vivo study). Single cell RNA-seq data are available in the NCBI's Gene Expression Omnibus under accession number GSE227251, https://www.ncbi.nlm.nih.gov/geo/query/acc.cgi?acc=GSE227251. The datasets used and analyzed during the study are available from the corresponding author on reasonable request.

Field-specific reporting

Please select the one below that is the best fit for your research. If you are not sure, read the appropriate sections before making your selection.

- [x] Life sciences
- [ ] Behavioural & social sciences
- [ ] Ecological, evolutionary & environmental sciences

For a reference copy of the document with all sections, see nature.com/documents/nr-reporting-summary-flat.pdf

Life sciences study design

All studies must disclose on these points even when the disclosure is negative.

Sample size	Due to the novelty of the hypothesis, power analysis is based on speculation and on preliminary data, pertinent to specific endpoints. For our in vivo studies, 10-15 animals for each timepoint are needed to detect a 20-30% effects of the interventions on functional or histological endpoints (power 0.90; alpha=0.05)
Data exclusions	No data were excluded from the analysis
Replication	Findings were replicated multiple times, as indicated by the sample size numbers, reflecting independent experiments.
Randomization	Allocation to experimental groups was random
Blinding	Investigators performing functional, histological or molecular analysis were blinded to the experimental conditions and to the genotype of the mice.

Reporting for specific materials, systems and methods

We require information from authors about some types of materials, experimental systems and methods used in many studies. Here, indicate whether each material, system or method listed is relevant to your study. If you are not sure if a list item applies to your research, read the appropriate section before selecting a response.

Materials & experimental systems

n/a	Involved in the study	
	x	Antibodies
x		Eukaryotic cell lines
x		Palaeontology and archaeology
	x	Animals and other organisms
x		Human research participants
x		Clinical data
x		Dual use research of concern

Methods

n/a	Involved in the study	
x		ChIP-seq
	x	Flow cytometry
x		MRI-based neuroimaging

Antibodies

Antibodies used

1.Anti-α-SMA antibody (Company: Sigma, Catalog Number: F3777, Clone: 1A4, Dilution: 1:150)
2.Anti-CD31 antibody (Company: Cell Signaling Technology, Catalog Number: 77699, Clone: D8V9E, Dilution: 1:100)
3.Anti-ITGA5 antibody (Company: Abcam, Catalog Number: ab150361, Clone: EPR7854, Dilution: 1:100 in immunofluorescence and 1:1000 in western blotting)
4.FITC Anti-GFP antibody (Company: Abcam, Catalog Number: ab6662, Clone: N/A, Dilution: 1:100)
5.Anti- Mac-2 antibody (Company: Cedarlane, Catalog Number: CL8942AP, Clone: M3/38, Dilution: 1:200)
6.Alexa Fluor 488 donkey anti-rabbit IgG (H+L) antibody (Company: Thermofisher, Catalog Number: A21206, Clone: N/A, Dilution:

1:500)
7.Alexa Fluor 594 donkey anti-rabbit IgG (H+L) antibody (Company: Thermofisher, Catalog Number: A21207, Clone: N/A, Dilution: 1:500)
8.Alexa Fluor 594 donkey anti-rat IgG (H+L) antibody (Company: Thermofisher, Catalog Number: A21209, Clone: N/A, Dilution: 1:500)
9.Anti- CD45-PE-Cy5 antibody (Company: BD Pharmingen, Catalog Number: 553082, Clone: 30-F11, Dilution: 1:1000)
10.Anti- CD11b-APC/Cyanine7 antibody (Company: Biolegend, Catalog Number: 101226, Clone: M1/70, Dilution: 1:1000)
11.Anti- Ly6G-PerCP/Cyanine5.5 antibody (Company: Biolegend, Catalog Number: 127616, Clone: 1A8, Dilution: 1:1000)
12.Anti- MerTK-PE antibody (Company: Biolegend, Catalog Number: 151506, Clone: 2B10C42, Dilution: 1:100)
13.Anti- CD64-FITC antibody (Company: Biolegend, Catalog Number: 139316, Clone: X54-5/7.1, Dilution: 1:100)
14.Anti- CD49e-APC antibody (Company: Biolegend, Catalog Number: 103814, Clone: 5H10-27, Dilution: 1:100)
15.Anti-Rat IgG2a, k Isotype Control-APC antibody (Company: Biolegend, Catalog Number: 400512, Clone: RTK2758, Dilution: 1:100)
16.Anti- CD16/32 antibody (Company: BD Pharmingen, Catalog Number: 553142, Clone: 2.4G2, Dilution: 1:100)
17.Anti- CD3e-APC antibody (Company: Biolegend, Catalog Number: 100311, Clone: 145-2C11, Dilution: 1:100)
18.Anti- p-AKT antibody (Company: Cell Signaling Technology, Catalog Number: 4060, Clone: D9E, Dilution: 1:1000)
19.Anti- AKT antibody (Company: Cell Signaling Technology, Catalog Number: 9272, Clone: N/A, Dilution: 1:1000)
20.Anti- p-P38 antibody (Company: Cell Signaling Technology, Catalog Number: 4511, Clone: D3F9, Dilution: 1:1000)
21.Anti- P38 antibody (Company: Cell Signaling Technology, Catalog Number: 8690, Clone: D13E1, Dilution: 1:1000)
22.Anti- p-ERK1/2 antibody (Company: Cell Signaling Technology, Catalog Number: 4370, Clone: D13.14.4E, Dilution: 1:1000)
23.Anti- ERK1/2 antibody (Company: Cell Signaling Technology, Catalog Number: 4695, Clone: 137F5, Dilution: 1:1000)
24.Anti- p-FAK antibody (Company: Cell Signaling Technology, Catalog Number: 3283, Clone: N/A, Dilution: 1:1000)
25.Anti- FAK antibody (Company: Cell Signaling Technology, Catalog Number: 3285, Clone: N/A, Dilution: 1:1000)
26.Anti- beta Actin antibody (Company: Cell Signaling Technology, Catalog Number: 4970, Clone: 13E5, Dilution: 1:1000)
27.Anti-rabbit IgG, HRP-linked antibody (Company: Cell Signaling Technology, Catalog Number: 7074, Clone: N/A, Dilution:1:2000)
28. Anti-ITGA5 antibody (Company: Biolegend, Catalog Number: 103910, Clone: HMα5-1, Dilution:1:100)
29. Anti-Armenian Hamster IgG Isotype Ctrl Antibody (Company: Biolegend, Catalog Number: 400902, Clone: HTK888, Dilution:1:50)

Validation

1. anti-α-SMA antibody(Sigma, F3777), we have extensively validated this antibody using paaraffin-embedded formalin-fixed mouse sections. Appropriate positive and negative controls were used (Shinde et al BBA Mol Bas Dis 2016). Additional validation provided in the manufacturer’s website(https://www.sigmaaldrich.cn/CN/zh/product/sigma/f3777).
2. anti-CD31 antibody(Cell Signaling Technology, #77699), the IHC stains of mouse small intestine, kidney, liver and A2058 xenograft using CD31 Rabbit mAb(Cell Signaling Technology, #77699) have been reported on the manufacturer website(https://www.cellsignal.com/products/primary-antibodies/cd31-pecam-1-d8v9e-xp-rabbit-mab/77699).
3. anti-ITGA5 antibody(Abcam, ab150361), 1).Immunofluorescence analysis of U937 (Human histiocytic lymphoma monocyte) cells using anti-ITGA5 antibody(Abcam, ab150361) has been reported on the manufacturer website; 2)WB analysis of mouse fibroblasts using anti-ITGA5 antibody(Abcam, ab150361) has been reported on the manufacturer website(https://www.abcam.cn/products/primary-antibodies/integrin-alpha-5-antibody-epr7854-ab150361.html).
4. anti-GFP antibody(Abcam, ab6662), immunofluorescence analysis of mouse brain tissue using anti-GFP antibody(Abcam, ab6662) has been reported on the manufacturer website(https://www.abcam.cn/products/primary-antibodies/fitc-gfp-antibody-ab6662.html).
5. anti-Mac-2 antibody(Cedarlane, CL8942AP), Thioglycolate-elicited C57BL/6 mouse peritoneal macrophages were stained with anti-Galectin-3, and 85.8 percentage of cells were stained positive above control IgG antibody(https://www.cedarlanelabs.com/products/detail/cl8942ap). We have validated the specificity of thus antibody using material from galectin-3 KO mice (Frunza et al Am J Pathol 2016).
6. Alexa Fluor 488 donkey anti-rabbit IgG (H+L) antibody(Thermofisher, A21206), immunofluorescence analysis of many tissues using this antibody has been reported on the manufacturer website(https://www.thermofisher.cn/cn/zh/antibody/product/Donkey-anti-Rabbit-IgG-H-L-Highly-Cross-Adsorbed-Secondary-Antibody-Polyclonal/A-21206).
7. Alexa Fluor 594 donkey anti-rabbit IgG (H+L) antibody(Thermofisher, A21207), immunofluorescence analysis of many tissues using this antibody has been reported on the manufacturer website(https://www.thermofisher.cn/cn/zh/antibody/product/Donkey-anti-Rabbit-IgG-H-L-Highly-Cross-Adsorbed-Secondary-Antibody-Polyclonal/A-21207).
8. Alexa Fluor 594 donkey anti-rat IgG (H+L) antibody(Thermofisher, A21209), immunofluorescence analysis of many tissues using this antibody has been reported on the manufacturer website(https://www.thermofisher.cn/cn/zh/antibody/product/Donkey-anti-Rabbit-IgG-H-L-Highly-Cross-Adsorbed-Secondary-Antibody-Polyclonal/A-21209).
9. Anti- CD45-PE-Cy5 antibody(BD Pharmingen, 553082), mouse splenic leucocytes were stained with anti- CD45-PE-Cy5 antibody(BD Pharmingen, 553082), https://www.bdbiosciences.com/en-us/products/reagents/flow-cytometry-reagents/research-reagents/single-color-antibodies-ruo/pe-rat-anti-mouse-cd45.553081.
10. Anti- CD11b-APC/Cyanine7 antibody(Biolegend, 101226), c57BL/6 mouse bone marrow stained with anti- CD11b-APC/Cyanine7 antibody(Biolegend, 101226), https://www.biolegend.com/en-us/products/apc-cyanine7-anti-mouse-human-cd11b-antibody-3930?GroupID=BLG10616.
11. Anti-Ly6G- PerCP/Cyanine5.5 antibody(BioLegend, 126715), c57BL/6 mouse bone marrow stained with anti-Ly6G- PerCP/Cyanine5.5 (BioLegend, 126715), https://www.biolegend.com/en-us/products/percp-cyanine5-5-anti-mouse-ly-6g-antibody-6116?GroupID=BLG7234).
12. Anti- MerTK-PE antibody(Biolegend, 151506), c57BL/6 mouse splenocytes were stained with anti-MERTK- PE antibody(Biolegend, 151506), https://www.biolegend.com/en-us/products/pe-anti-mouse-mertk-mer-antibody-13406?GroupID=BLG15085.
13. Anti- CD64-FITC antibody(Biolegend, 139316), c57BL/6 mouse bone marrow cells were stained with anti-CD64-FITC antibody (Biolegend, 139316), https://www.biolegend.com/en-us/products/fitc-anti-mouse-cd64-fcgammari-antibody-12422?GroupID=BLG8805.
14. Anti- CD49e-APC antibody(Biolegend, 103814), c57BL/6 mouse bone marrow cells were stained with anti-CD49e-APC antibody (Biolegend, 103814), https://www.biolegend.com/nl-be/products/apc-anti-mouse-cd49e-antibody-16202?GroupID=BLG2427.
15. Anti-Rat IgG2a, k Isotype Control-APC antibody(Biolegend, 400512), the validation information has been reported on the manufacturer website(https://www.biolegend.com/en-us/products/apc-rat-igg2a-kappa-isotype-ctrl-1838).
16. anti-CD16/32 antibody (BD Pharmingen, 553142), two color analysis of the expression of CD16/CD32 on mouse spleen cells and demonstration of FCγR-mediated non-specific staining by anti-CD16/32 antibody in flow(BD Pharmingen, 553142)(https://

www.bdbiosciences.com/en-us/products/reagents/flow-cytometry-reagents/research-reagents/single-color-antibodies-ruo/purified-rat-anti-mouse-cd16-cd32-mouse-bd-fc-block.553142).
17. Anti- CD3e-APC antibody(Biolegend, 100311), c57BL/6 mouse splenocytes were stained with Anti- CD3e-APC antibody(Biolegend, 100311), https://www.biolegend.com/en-us/products/apc-anti-mouse-cd3epsilon-antibody-21.
18. anti-p-AKT antibody(Cell Signaling Technology, 4060), WB analysis of different cell types using anti-p-AKT antibody(Cell Signaling Technology, 4060) has been reported on the manufacturer website (https://www.cellsignal.com/products/primary-antibodies/phospho-akt-ser473-d9e-xp-rabbit-mab/4060).
19. anti-AKT antibody(Cell Signaling Technology, 9272), WB analysis of different cell types using anti-AKT antibody(Cell Signaling Technology, 9272)has been reported on the manufacturer website(https://www.cellsignal.com/product/productDetail.jsp?productId=9272).
20. anti-p-P38 antibody(Cell Signaling Technology, 4511), WB analysis of different cell types using anti-p-P38 antibody(Cell Signaling Technology, 4511) has been reported on the manufacturer website(https://www.cellsignal.com/product/productDetail.jsp?productId=4511).
21. anti-P38 antibody (Cell Signaling Technology, 8690), WB analysis of different cell types using anti-P38 antibody (Cell Signaling Technology, 8690) has been reported on the manufacturer website(https://www.cellsignal.com/product/productDetail.jsp?productId=8690).
22. anti-p-ERK1/2 antibody(Cell Signaling Technology, 4370), WB analysis of different cell types using anti-p-ERK1/2 antibody(Cell Signaling Technology, 4370) has been reported on the manufacturer website(https://www.cellsignal.com/product/productDetail.jsp?productId=4370).
23. anti-ERK1/2 antibody(Cell Signaling Technology, 4695), WB analysis of different cell types using anti-ERK1/2 antibody(Cell Signaling Technology, 4695) has been reported on the manufacturer website(https://www.cellsignal.com/product/productDetail.jsp?productId=4695).
24. anti-p-FAK antibody (Cell Signaling Technology, 3283), WB analysis of different cell types using anti-p-FAK antibody (Cell Signaling Technology, 3283) has been reported on the manufacturer website(https://www.cellsignal.com/product/productDetail.jsp?productId=3283).
25. anti-FAK antibody(Cell Signaling Technology, 3285), WB analysis of different cell types using anti-FAK antibody(Cell Signaling Technology, 3285) has been reported on the manufacturer website(https://www.cellsignal.com/product/productDetail.jsp?productId=3285).
26. anti-beta Actin antibody (Cell Signaling Technology, 4970), WB analysis of different cell types using anti-beta Actin antibody (Cell Signaling Technology, 4970) has been reported on the manufacturer website(https://www.cellsignal.com/product/productDetail.jsp?productId=4970).
27. nnti-rabbit IgG, HRP-linked antibody(Cell Signaling Technology, 7074), the validation information has been reported on the manufacturer website(https://www.cellsignal.com/products/secondary-antibodies/anti-rabbit-igg-hrp-linked-antibody/7074).
28. anti-ITGA5 antibody (Biolegend, 103910), c57BL/6 mouse bone marrow cells stained with anti-ITGA5 antibody (Biolegend, 103910), followed by anti-Armenian hamster IgG FITC, https://www.biolegend.com/en-us/products/ultra-leaf-purified-anti-mouserat-cd49e-antibody-19111.
29. Anti-Armenian Hamster IgG Isotype Ctrl Antibody (Biolegend, 400902), IP analysis of Jurkat cells using Anti-Armenian Hamster IgG Isotype Ctrl Antibody (Biolegend, 400902) has been reported on the manufacturer website(https://www.biolegend.com/en-us/products/purified-armenian-hamster-igg-isotype-ctrl-1780?GroupID=BLG2376).

Animals and other organisms

Policy information about studies involving animals; ARRIVE guidelines recommended for reporting animal research

Laboratory animals	Mice, C57Bl6J background, both male and female.<br>CX3CR1Cre-ER;ITGA5fl/fl vs CX3CR1-CreER vs ITGA5 fl/fl<br>LysMCre;ITGA5 fl/fl vs LysMCre vs ITGA5 fl/fl<br>CSF1R-EGFP
Wild animals	The study did not involve wild animals
Field-collected samples	The study did not involve field-collected samples
Ethics oversight	All animal studies were approved by the Institutional Animal Care and Use Committee at Albert Einstein College of Medicine.

Note that full information on the approval of the study protocol must also be provided in the manuscript.

Flow Cytometry

Plots

Confirm that:

- [x] The axis labels state the marker and fluorochrome used (e.g. CD4-FITC).
- [x] The axis scales are clearly visible. Include numbers along axes only for bottom left plot of group (a 'group' is an analysis of identical markers).
- [x] All plots are contour plots with outliers or pseudocolor plots.
- [x] A numerical value for number of cells or percentage (with statistics) is provided.

Methodology

Sample preparation	Male and female WT C57Bl6/J mice underwent non-reperfused infarction protocols (7 or28 days of coronary occlusion). Sham controls underwent thoracotomy without coronary occlusion. Mice were sacrificed and intracardially perfused with 25 mL of ice-cold phosphate-buffered saline to remove blood cells. Whole hearts were quickly minced and digested by shaking the tissue fragments (15 min at 37°C water bath, repeated 3 times) in 3.0 ml of digestion buffer (RPMI 1640-medium (Sigma-Aldrich) containing 120 mg/ml of Liberase TH (Roche, 5401151001) and 70 mg/ml of DNase I (Roche, 10104159001). After digestion, single cell suspensions were harvested by passing through a 30-μm cell strainer and by washing with ice-cold phosphate-buffered saline.
Instrument	Cytek AURORA
Software	SpectroFlo (Cytek Biosciences)<br>FlowJo_v10.8.0 (BD Biosciences)
Cell population abundance	After doublets were excluded by FSC-H vs. FSC-A, DAPI-negative live cells were identified. Live cells (DAPI- cells) accounted for 82.8-93.0% of the doublet-depleted population. Myeloid cells were identified as DAPI-/CD45+/CD11b+ cells. Myeloid cells accounted for 2-3.5% in Sham mice, 19.4-51.8% in MI day7 mice, and 2.3-7.9% in MI day28 mice of live cells (DAPI- cells). Macrophages were identified as DAPI-/CD45+/CD11b+/Ly6G-/CD64+/MerTK+ cells. Macrophages accounted for 0.5-1.2% in Sham mice, 6.9-23.4% in MI day7 mice, and 0.6-2.6% in MI day28 mice of live cells (DAPI- cells). T cells were identified as DAPI-/CD45+/CD11b-/CD3e+ cells. We only evaluated T cells at 7 days after MI, and T cells accounted for 0.3-1.3% in MI day7 mice of live cells (DAPI- cells).
Gating strategy	After doublets were excluded by FSC-H vs. FSC-A, DAPI-negative live cells were identified. Myeloid cells were identified as DAPI- CD45+ CD11b+ cells. After exclusion of DAPI- CD45+ CD11b+ Ly6G+ cells (neutrophils), macrophages were identified as DAPI- CD45+ CD11b+ Ly6G- CD64+ MerTK+ cells. T cells were identified as DAPI-/CD45+/CD11b-/CD3e+ cells. Positive and negative cells could be clearly distinguished for DAPI, CD45, CD11b, and Ly6G,

☒ Tick this box to confirm that a figure exemplifying the gating strategy is provided in the Supplementary Information.
